# Targeting Netrin-1 Mediates the Suppression of Osteolytic Bone Metastasis in Breast Cancer by Ugonin L

**DOI:** 10.7150/ijbs.128973

**Published:** 2026-06-10

**Authors:** Trung-Loc Ho, Wei-Cheng Chen, Chun-Lin Liu, Kuan-Ying Lai, Chih-Chuang Liaw, Lan-Yun Chang, Chih-Hao Lu, Hsiao-Chi Tsai, Yi-Chin Fong, Jeng-Hung Guo, Tzu-Min Huang, Chih-Ying Wu, Chih-Hsin Tang

**Affiliations:** 1 Department of Pharmacology, School of Medicine, China Medical University, Taichung, Taiwan.; 2 CirTech Institute, HUTECH University, Ho Chi Minh City, Vietnam.; 3 Department of Medicine, MacKay Medical University, New Taipei, Taiwan.; 4 Division of Sports Medicine & Surgery, Department of Orthopedic Surgery, MacKay Memorial Hospital, Taipei, Taiwan.; 5 Department of Neurosurgery, China Medical University Hospital, Taichung, Taiwan.; 6 Department of Marine Biotechnology and Resources, National Sun Yat-sen University, Kaohsiung, Taiwan.; 7 Graduate Institute of Natural Products, Kaohsiung Medical University, Kaohsiung, Taiwan; 8 Institute of Bioinformatics and Systems Biology, National Yang Ming Chiao Tung University, Hsinchu, Taiwan.; 9 Center for Intelligent Drug Systems and Smart Bio-Devices (IDS 2 B), National Yang Ming Chiao Tung University, Hsinchu, Taiwan.; 10 Department of Medicine Research, China Medical University Beigang Hospital, Yunlin, Taiwan.; 11 Department of Sports Medicine, College of Health Care, China Medical University, Taichung, Taiwan.; 12 Department of Orthopedic Surgery, China Medical University Hospital, Taichung, Taiwan.; 13 Department of Orthopedic Surgery, China Medical University Beigang Hospital, Yunlin, Taiwan.; 14 Department of Chest Surgery, China Medical University Hospital, Taichung, Taiwan.; 15 School of Chinese Medicine, China Medical University, Taichung, Taiwan.; 16 Department of Neurosurgery, China Medical University Hsinchu Hospital, Hsinchu, Taiwan.; 17 Chinese Medicine Research Center, China Medical University, Taichung, Taiwan.; 18 Department of Medical Laboratory Science and Biotechnology, College of Medical and Health Science, Asia University, Taichung, Taiwan.

**Keywords:** Ugonin L, breast cancer, osteolytic bone metastasis, Netrin-1

## Abstract

Over 70% of patients experience bone metastases, a serious and crippling side effect of breast cancer that increases morbidity. There is an urgent need for innovative therapies because current therapeutic options, including systemic and local treatments, are limited by significant side effects and poor survival improvements. *Helminthostachys zeylanica* is the source of Ugonin L, a naturally occurring substance with established anti-inflammatory properties. However, its role in breast cancer-associated osteolytic bone metastasis remains elusive. Here, we demonstrate that Ugonin L suppresses epithelial-mesenchymal transition (EMT), migration, and invasion of breast cancer cells. We identify Netrin-1 (NTN-1) as a critical mediator of Ugonin L-induced inhibition of EMT and cell motility. Clinical data analyses revealed that elevated NTN-1 expression is significantly linked with disease progression, poor overall survival, and bone metastasis in breast cancer patients. Mechanistically, Ugonin L inhibits NTN-1-dependent EMT and motility by suppressing the canonical Wnt/β-catenin signaling pathway. Furthermore, Ugonin L attenuates breast cancer-promoted osteoclast differentiation by downregulating NTN-1 expression. Importantly, Ugonin L markedly suppresses breast cancer-induced osteolytic bone lesions *in vivo*. Collectively, these results position Ugonin L as a promising therapeutic candidate for the prevention and remedy of osteolytic bone metastasis in breast cancer.

## 1. Introduction

Breast cancer is the most commonly diagnosed malignancy in women, with incidence continuing to rise worldwide and over 316,000 new cases anticipated in the United States in 2025, yet it remains the second leading cause of cancer-associated mortality in women after lung cancer 1. A defining feature of advanced breast cancer is its marked propensity for bone metastasis, which develops in up to 80% of patients with distant disease and results in skeletal complications that substantially limit survival and impair quality of life 2, 3. Patients with breast cancer bone metastases had a median survival of 19 months, and the five-year survival rate was as low as 13% 4, 5. The majority of breast cancer bone metastases are osteolytic, with intricate tumor-bone interactions that support tumor growth, skeletal degradation, and resistance to treatment. These metastases can result in serious side effects such compression of the spinal cord, discomfort, hypercalcemia, and fractures 6. The urgent need for additional effective therapies is highlighted by the fact that current therapeutic approaches, such as radiation, chemotherapy, endocrine therapy, and surgery, are mostly palliative and do not completely prevent skeletal problems or disease recurrence.

Disseminated tumor cells must overcome multiple environmental obstacles to create additional tumors, making metastatic colonization a deadly occurrence 7, 8. Epithelial-mesenchymal transition (EMT) is a key pathway through which malignant epithelial cells acquire a mesenchymal phenotype, gaining invasive, metastatic and bone metastatic properties 9, 10. Netrin-1 (NTN-1), located on chromosome 17p13.1, encodes a laminin-related protein essential for axon guidance and cell migration during nervous system development 11, 12. Beyond its neurobiological role, aberrant NTN-1 expression has been implicated in tumor progression, where it promotes EMT and metastatic dissemination, underscoring its potential as a prognostic and pharmacological target 13, 14. Although NTN-1 is well established as a regulator of EMT, its role in osteoclastogenesis within the bone metastatic niche remains largely undefined, highlighting NTN-1 as a potential therapeutic target for mitigating osteolytic bone metastasis.

Effective therapy for osteolytic bone metastasis must target both tumor progression and skeletal integrity. Bisphosphonates reduce skeletal complications 15, and denosumab, a monoclonal antibody targeting RANKL, was developed with similar aims; however, a recent phase III trial showed no significant improved overall survival benefit 16, 17. Current treatment strategies remain largely palliative, with limited efficacy and notable adverse effects 18, 19. Ugonin is a prenylated flavonoid isolated from the rhizomes of *Helminthostachys zeylanica* (L.) Hook. (Ophioglossaceae), a medicinal fern traditionally used in East and Southeast Asia 20. As one of the major bioactive constituents of this plant, Ugonin L has been reported to exhibit diverse pharmacological functions, such as antioxidant, anti-inflammatory, anti-osteoclastogenic, and anti-cancer effects 21-23. Recent studies have highlighted its potential role in modulating key cellular pathways associated with bone and joint disorders 24-26, suggesting that ugonin L represents a promising natural compound for the development of novel therapeutic strategies, thereby underscoring its potential clinical relevance. Here, we demonstrate that Ugonin L exerts potent anti-metastatic effects by inhibiting NTN-1-dependent EMT and motility in breast cancer cells through suppression of the Wnt/β-catenin signaling pathway. Moreover, Ugonin L effectively attenuates breast cancer-induced osteoclast formation and osteolytic bone destruction both *in vitro* and *in vivo*. These findings highlight Ugonin L as a promising therapeutic candidate for the remedy of breast cancer osteolytic bone metastasis.

## 2. Material and Methods

### 2.1 Reagent and materials

Ugonin L was provided by Dr. Chih-Chuang Liaw (National Sun Yat-sen University, Taiwan) and isolated from the methanol extract of *Helminthostachys zeylanica* rhizomes using repeated chromatographic methods 21. GSK-3 inhibitor IX (BIO; 6-bromoindirubin-3′-oxime; CAS No. 667463-62-9), a Wnt/β-catenin pathway activator, was purchased from Sigma-Aldrich (St. Louis, MO, USA). NTN-1 plasmids were synthesized by MDBio, Inc. (Taiwan). The human NTN-1 ELISA kit (Cat. No. NBP2-76771) was obtained from Novus Biologicals, and the mouse NTN-1 ELISA kit (Cat. No. MBS453734) was obtained from MyBioSource. Antibodies against NTN-1 (SC-518135, Santa Cruz), N-Cadherin (ab76057, Abcam), E-cadherin (ab40772, Abcam), ZO-1 (GTX108592, Genetex), pGSK3β (SC-81496, Santa Cruz), GSK3β (SC-9166, Santa Cruz), β-Catenin (SC-133240, Santa Cruz), and β-Actin (A5441, Sigma-Aldrich) were used.

Detailed experimental procedures for the MTT assay, Boyden chamber migration and invasion assays, real-time quantitative PCR (qPCR), Western blotting, nuclear and cytoplasmic fractionation, immunofluorescence staining, ELISA, and immunohistochemical (IHC) staining, as well as primer sequences and antibody working concentrations, are provided in the Supplementary data.

### 2.2 Cell cultures

The human breast cancer cell lines MDA-MB-231, MCF-7, T-47D, and BT-474 as well as the murine breast cancer cell line 4T1, were obtained from the American Type Culture Collection (ATCC, Manassas, VA, USA). The murine macrophage cell line RAW264.7 was obtained from the Bioresource Collection and Research Center (BCRC, Hsinchu, Taiwan). MDA-MB-231 cells were cultured in Dulbecco's Modified Eagle Medium (DMEM; Gibco, USA), MCF-7 cells were maintained in DMEM/F-12 medium (Gibco, USA), and T-47D and 4T1 cells were cultured in RPMI-1640 medium (Gibco, USA). T-47D cells were additionally supplemented with insulin (10 μg/mL; Sigma-Aldrich, USA). RAW264.7 cells were maintained in DMEM (Gibco, USA). The culture medium was supplemented with 10% fetal bovine serum (FBS) and 1% penicillin-streptomycin (100 U/mL penicillin and 100 μg/mL streptomycin), both sourced from Gibco (USA). Cells were incubated at 37 °C in a humidified atmosphere containing 5% CO₂. Cells (2 × 10⁶) were seeded and allowed to adhere for 24 h until reaching an appropriate confluency (approximately 70-80%) before drug treatment. Cells in the logarithmic growth phase were then used for subsequent experiments.

### 2.3 Bioinformatic analysis

The METS cancer metastasis database (http://biocc.hrbmu.edu.cn/METS) was employed to assess EMT scores in breast cancer. The expression level of *NTN-1* in breast cancer and normal tissues was analyzed using data from The Cancer Genome Atlas (TCGA). In addition, the UALCAN web resource (http://ualcan.path.uab.edu) was used to further evaluate NTN-1 expression across normal and tumor samples of breast invasive carcinoma patients.

### 2.4 Kaplan-Meier plotter survival analysis

The correlation between *NTN-1* gene expression and overall survival (OS) in breast cancer patients was evaluated using the Kaplan-Meier plotter database (http://kmplot.com/analysis). Patients were stratified into high and low expression groups based on the median expression of *NTN-1*gene, and survival curves were generated using the Kaplan-Meier method. Hazard ratios (HR) with 95% confidence intervals (CIs) and log-rank p values were calculated to assess statistical significance.

### 2.5 Human Protein Atlas analysis

The Human Protein Atlas (HPA; https://www.proteinatlas.org/) was used to evaluate the protein expression of NTN-1 in normal breast tissues (n = 2) and breast cancer patient tissues (n = 9) through IHC data. Representative images and expression levels were compared between normal and tumor samples to assess NTN-1 expression patterns.

### 2.6 Gene Expression Omnibus analysis

The GSE22820 and GSE46141 dataset was obtained from the Gene Expression Omnibus (GEO) database (https://www.ncbi.nlm.nih.gov/geo/). This dataset contains transcriptomic profiles of primary breast cancer tissues and breast cancer bone metastasis samples, generated using the [GPL6480 and GPL10379] Affymetrix Human Gene 1.0 ST Array platform. Data were processed and normalized as provided by GEO. Differential expression analysis between primary breast cancer and bone metastatic tissues was conducted using GEO2R with the limma package, applying thresholds of *p* < 0.05 and |log₂ fold change| > 1. Expression levels of *NTN-1* gene signatures were specifically examined to assess their association with osteolytic bone metastasis in breast cancer.

### 2.7 Ingenuity pathway analysis

Differentially expressed genes (DEGs) derived from transcriptomic datasets were analyzed using Ingenuity Pathway Analysis (IPA, QIAGEN Inc., Redwood City, CA, USA). Data was uploaded into IPA, and Core Analysis was performed to identify enriched canonical signaling pathways, upstream regulators, diseases and biological functions, and molecular interaction networks. The statistical significance of pathway enrichment was evaluated using a right-tailed Fisher's exact test, with *p* < 0.05 as the cutoff. The activation z-score was applied to predict the activation or inhibition state of specific biological functions and upstream regulators. Visualization of pathway networks and heatmaps was generated using the IPA platform to interpret the functional roles of DEGs in breast cancer progression and metastasis.

### 2.8 SRPlot and KEGG pathway analysis

DEGs were analyzed using SRPlot (http://www.bioinformatics.com.cn), an online bioinformatics visualization platform, to generate volcano plots, heatmaps, and bubble charts for intuitive data presentation. For functional annotation, DEGs were subjected to KEGG pathway enrichment analysis using the cluster Profiler R package.

### 2.9 Single cell RNA-analysis

The dataset was processed to assess *NTN-1* gene expression across cell populations. Single-cell RNA sequencing (scRNA-seq) data were obtained from the Gene Expression Omnibus database (accession number: GSE161529 and GSE190772) were analyzed to evaluate *NTN-1* expression across diverse cellular populations. Raw count matrices were generated using CellRanger (10x Genomics, version 2.0), followed by standard quality control procedures-excluding genes detected in fewer than three cells and cells with under 200 expressed genes. Cells exhibiting > 20% mitochondrial gene content were filtered out to remove low-quality or apoptotic cells. Data were normalized and variance-stabilized using the SCTransform method, followed by principal component analysis (PCA). Datasets were integrated using Seurat's anchor-based workflow with the top 2,000 variable features and the first 30 principal components to correct for batch effects. The integrated data were then clustered using a shared nearest-neighbor graph and the Louvain algorithm. Cluster identities were assigned based on differential gene expression analysis using Scanpy's rank_genes_groups function, with significant markers defined as an adjusted p value < 0.05 and log₂ fold change > 0.5. Clusters were annotated using canonical marker genes. t-distributed stochastic neighbor embedding (t-SNE) was used for visualization. Finally, *NTN-1* expressions were examined across annotated cell types and experimental conditions.

### 2.10 Molecular Docking

Molecular docking was performed using SwissDock (http://www.swissdock.ch/) to predict the binding interactions between ligands and target proteins. The three-dimensional structure of human NTN-1 was retrieved from the Protein Data Bank (PDB ID: 4URT), and the chemical structure of Ugonin L was obtained from the PubChem database (CID: 10365741). The resulting docking poses were analyzed and visualized using Discovery Studio Visualizer (BIOVIA, Dassault Systèmes, San Diego, CA, USA).

### 2.11 TRAP staining

Conditioned medium (CM) preparation: MDA-MB-231 and 4T1 cells (2 × 10⁶ cells) were seeded in 100-mm culture dishes and cultured until reaching approximately 80-90% confluence. The cells were then incubated in culture medium containing 1% FBS, with or without the indicated treatments for 72 h. Following incubation, the conditioned medium was collected and centrifuged to remove cellular debris, and the clarified supernatant was subsequently used for osteoclast differentiation assays 27. RAW264.7 cells (5000 cells/well) were cultured in α-MEM supplemented with 10% FBS and induced to differentiate into osteoclasts in the presence or absence of breast cancer cell-CM for 5 days 28. The medium was replaced every 2 days, and cells were subsequently stained using a tartrate-resistant acid phosphatase (TRAP) staining kit (Sigma-Aldrich, St. Louis, MO, USA). Bone cross-section slides were also subjected to TRAP staining following the manufacturer's instructions.

### 2.12 Osteolytic bone metastasis animal model

To establish the breast cancer-induced bone metastasis model, 4T1-GFP-Luc cells (2 × 10⁵) were injected into the caudal artery of six-week-old female BALB/c mice (BioLASCO Taiwan Co., Ltd., Taipei, Taiwan). Following tumor induction, mice were randomly divided into four groups (n = 8 per group): normal group (without tumor cell injection), control group (4T1 + vehicle), low-dose treatment group, and high-dose treatment group. The vehicle consisted of DMSO at a final concentration of 0.1% (v/v). Mice in the treatment groups received Ugonin L at doses of 5 mg/kg (low dose) or 15 mg/kg (high dose). The high dose (15 mg/kg) corresponds to approximately 300 μg per mouse, which is equivalent to approximately 33-43 mg of *Helminthostachys zeylanica* extract per mouse, based on a reported content of 7-9 mg Ugonin L per gram of extract. After 4 weeks, bioluminescent signals were detected using the IVIS® Spectrum imaging system to monitor tumor development and distribution *in vivo*. Subsequently, all animals were humanely euthanized, and tumor-associated bone destruction was evaluated by X-ray and micro-computed tomography (micro-CT) imaging. Bone specimens were collected for histological analysis, including hematoxylin and eosin (H&E) staining, and examined under a light microscope for morphological changes. All experimental procedures were approved by the Institutional Animal Care and Use Committee (IACUC) of China Medical University.

### 2.13 Statistical analysis

All statistical analyses were performed using GraphPad Prism software (version 8.2; GraphPad Software, San Diego, CA, USA). Prior to applying parametric tests, data distribution was assessed for normality using the Shapiro-Wilk test, and homogeneity of variance was evaluated using Levene's test. Comparisons among more than three groups were conducted using one-way analysis of variance (ANOVA) followed by Bonferroni's post hoc test. For experiments involving two or more independent variables, two-way ANOVA was applied. For comparisons between two groups, a Student's *t*-test was applied. Data are presented as the mean ± standard deviation (SD), and a value of *p* < 0.05 was considered statistically significant.

## 3. Results

### 3.1 Ugonin L inhibits motility and EMT in breast cancers cells

**Figure 1:**
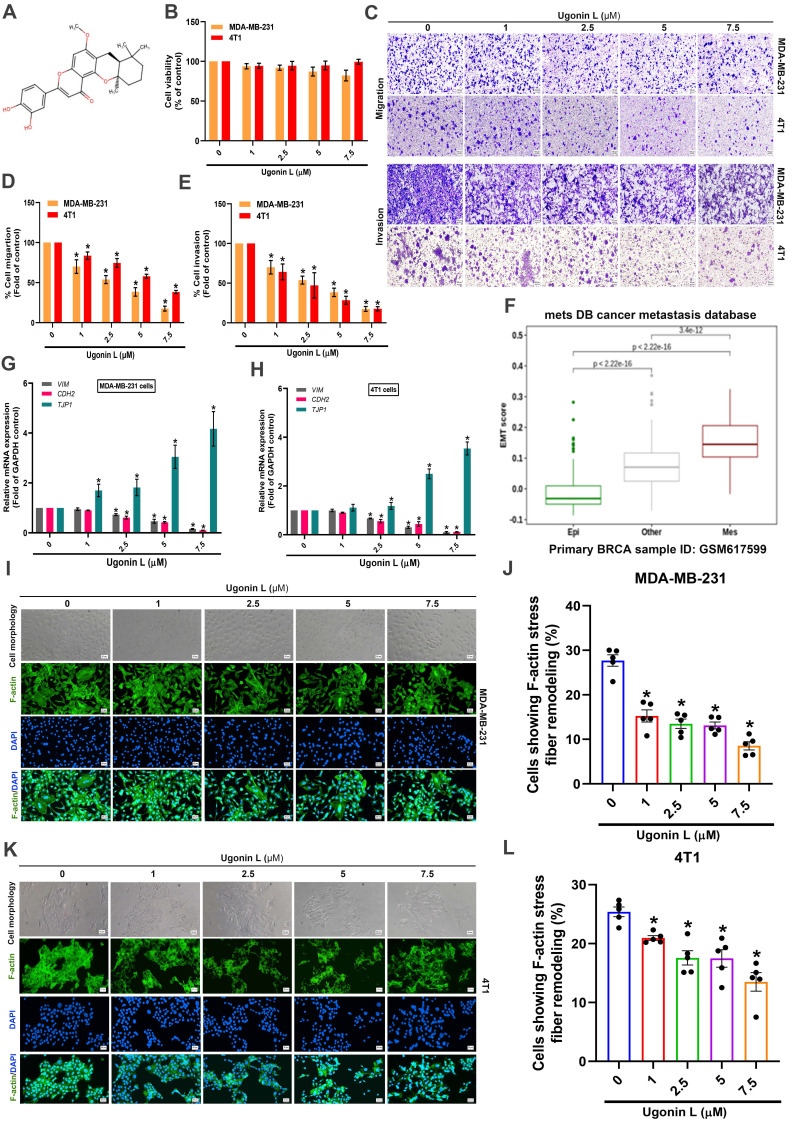
** Ugonin L inhibits breast cancer cell migration and invasion without affecting cell viability and modulates EMT-associated properties in breast cancer cells.** (A) Chemical structure of Ugonin L. (B) Cell viability of MDA-MB-231 and 4T1 breast cancer cells treated with Ugonin L (1-7.5 μM) for 24 h, measured by MTT assay. (C-E) Migration and invasion assays evaluating the migratory and invasive abilities of breast cancer cells following treatment with Ugonin L at various concentrations (1-7.5 μM) for 24 h, including representative images and quantitative analyses. (F) The EMT score in breast cancer patients were analyzed using the MetsDB database. (G&H) Cells were treated with Ugonin L for 24 h, the mRNA expression of indicated EMT markers was examined by qPCR. (I-L) Cells were treated with Ugonin L for 24 h, and F-actin organization was examined by immunofluorescence staining. Quantification of F-actin stress fiber remodeling was performed using ImageJ software. Scale bar = 100 μm. Results are presented as the mean ± SD of three independent biological experiments (n = 3). *p < 0.05 compared with the control group.

To evaluate the effects of Ugonin L (Figure 1A) on breast cancer cell behavior, we first assessed cell viability using an MTT assay. The results showed that Ugonin L exhibited no significant cytotoxicity in breast cancer cells (MDA-MB-231, 4T1, MCF-7, T-47D) (Figure 1B; Supplementary Figure 1A). Nevertheless, cell migration and invasion were markedly reduced in a concentration-dependent manner following Ugonin L treatment (Figure 1C-E; Supplementary Figure 1B-E), suggesting that Ugonin L suppresses breast cancer cell motility without affecting viability. Analysis using the MetsDB database indicated that the EMT score was significantly elevated in human breast cancer, accompanied by upregulation of mesenchymal markers (Figure 1F). Since MDA-MB-231 and 4T1 cells are commonly used as triple-negative breast cancer (TNBC)-like models, we further confirmed their receptor-negative phenotype by western blotting. Both cell lines showed undetectable or low expression of ER, PR, and HER2, together with low basal E-cadherin expression, supporting their suitability for investigating EMT and metastatic behavior in TNBC-like breast cancer cells (Supplementary Figure 2). qPCR analysis revealed that Ugonin L treatment suppressed EMT by downregulating mesenchymal markers *VIM* (Vimentin) and *CDH2* (N-cadherin) and upregulating epithelial markers *TJP1* (ZO-1) in both MDA-MB-231 and 4T1 cells (Figure 1G&H). Abnormal remodeling of filamentous actin (F-actin) is associated to EMT and metastatic progression in various cancers, including breast cancer 29-31. Enhanced F-actin dynamics is widely regarded as a characteristic feature of aggressive tumor cells, which generate specialized actin-rich structures such as filopodia to promote migration and invasion 32. In the present study, control cells displayed a more elongated, spindle-like morphology with prominent stress fibers, whereas Ugonin L-treated cells exhibited a more rounded morphology and reduced stress fiber formation, indicating altered cytoskeletal organization (Figure 1I-L). These observations suggest that Ugonin L attenuates EMT-associated cytoskeletal remodeling and cell motility in breast cancer cells.

### 3.2 Ugonin L suppresses EMT and motility in breast cancer cells by inhibiting NTN-1 expression

**Figure 2:**
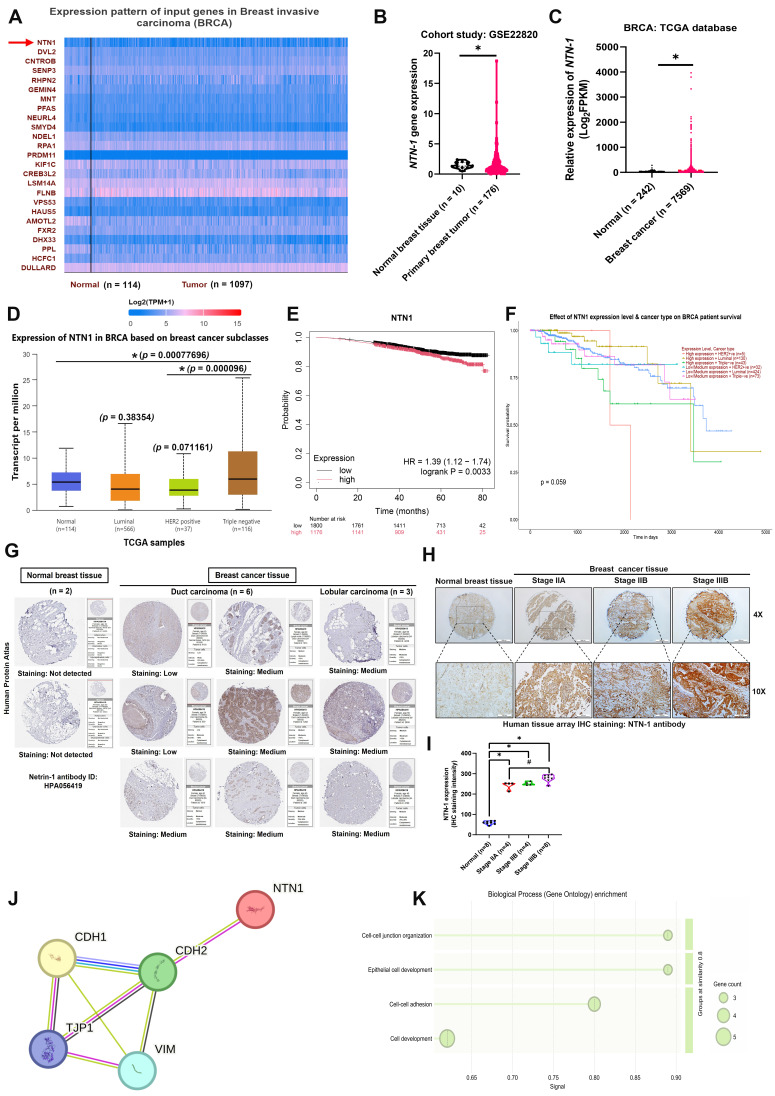
** NTN-1 expression and clinical relevance in breast cancer.** (A) Heatmap analysis of gene expression patterns in breast invasive carcinoma (BRCA) from the TCGA dataset with *NTN-1* highlighted (BRCA cohort: n = 1097 tumor vs. n = 114 normal). (B&C) NTN-1 mRNA expression levels in normal breast tissues and primary breast tumors from the GSE22820 cohort (n = 176 tumors vs. n = 10 normal tissues) and the TCGA transcriptomic dataset (n = 7569 tumors vs. n = 242 normal tissues). (D) *NTN-1* expression in normal (n = 114), and across different breast cancer molecular subtypes, including luminal (n = 556), HER2-positive (n = 37), and triple-negative breast cancer (TNBC) (n = 116). (E&F) Kaplan-Meier survival analysis of overall survival in BRCA patients stratified by *NTN-1* expression levels, and survival analysis based on NTN-1 expression across different breast cancer subtypes. (G) Representative IHC staining images of NTN-1 protein expression in normal breast tissue and breast cancer tissues obtained from the Human Protein Atlas database (normal tissues, n = 2; tumor tissues, n = 9). (H&I) Representative IHC staining of NTN-1 protein in human breast tissue arrays (Serial ID: BR087-L56), including normal tissue (n = 8) and different tumor stages [Stage IIA (n = 4), Stage IIB (n = 4), and Stage IIIB (n = 4)], along with quantification of NTN-1 staining intensity. (J) Interaction network analysis showing the association of *NTN-1* with EMT-related markers, including *CDH1*, *CDH2*, *VIM*, and *TJP1*. (K) Gene Ontology (GO) biological process enrichment analysis of *NTN-1*-associated genes. Data are presented as mean ± SD. *p < 0.05 compared with the indicated groups.

NTN-1 has been reported to play a key role in EMT and tumor metastasis 33. Based on previous evidence that NTN-1 is functionally involved in both tumor progression and osteoclast-related metastasis 34-36. On this basis, we considered NTN-1 a potential mediator linking EMT, breast cancer cell motility, and osteolytic bone metastasis. Therefore, to further elucidate the mechanism of Ugonin L, we investigated whether its inhibitory effects on EMT, cell motility, and osteolytic bone metastasis were associated with modulation of NTN-1 expression. Analysis of the ULCAN dataset revealed that *NTN-1* expression was markedly elevated in breast cancer tissues compared with normal controls, as shown by heatmap profiling (Figure 2A). This upregulation was further confirmed in independent datasets, including the GSE22820 cohort and TCGA database, where *NTN-1* expression was significantly higher in tumor tissues than in normal breast tissues (Figure 2B&C). Subgroup analysis demonstrated that *NTN-1* expression was particularly increased in triple-negative breast cancer (TNBC) compared with other molecular subtypes (Figure 2D). Kaplan-Meier survival analysis showed that elevated *NTN-1* expression was associated with poorer overall survival in breast cancer patients (Figure 2E) and across different breast cancer subtypes (Figure 2F), suggesting its potential prognostic relevance.

Consistently, immunohistochemical data from the Human Protein Atlas (HPA) showed low or undetectable NTN-1 protein expression in normal breast tissues but moderate to high expression in breast cancer tissues (Figure 2G). Moreover, tissue microarray analysis further demonstrated that NTN-1 protein expression increased with tumor progression, with significantly higher staining intensity observed in advanced-stage breast cancer tissues compared with normal tissues (Figure 2H&I). Mechanistically, protein-protein interaction (PPI) network analysis revealed that *NTN-1* interacts with key EMT-related markers, including *CDH1*, *CDH2*, *VIM*, and *TJP1* (Figure 2J). Gene Ontology (GO) enrichment analysis further indicated that *NTN-1*-associated genes are involved in biological processes related to cell-cell junction organization, epithelial cell development, and cell adhesion, which are closely linked to EMT and tumor cell motility (Figure 2K). Collectively, these findings demonstrate that NTN-1 is upregulated in breast cancer, correlates with poor prognosis and tumor progression, and is functionally associated with EMT-related pathways.

**Figure 3:**
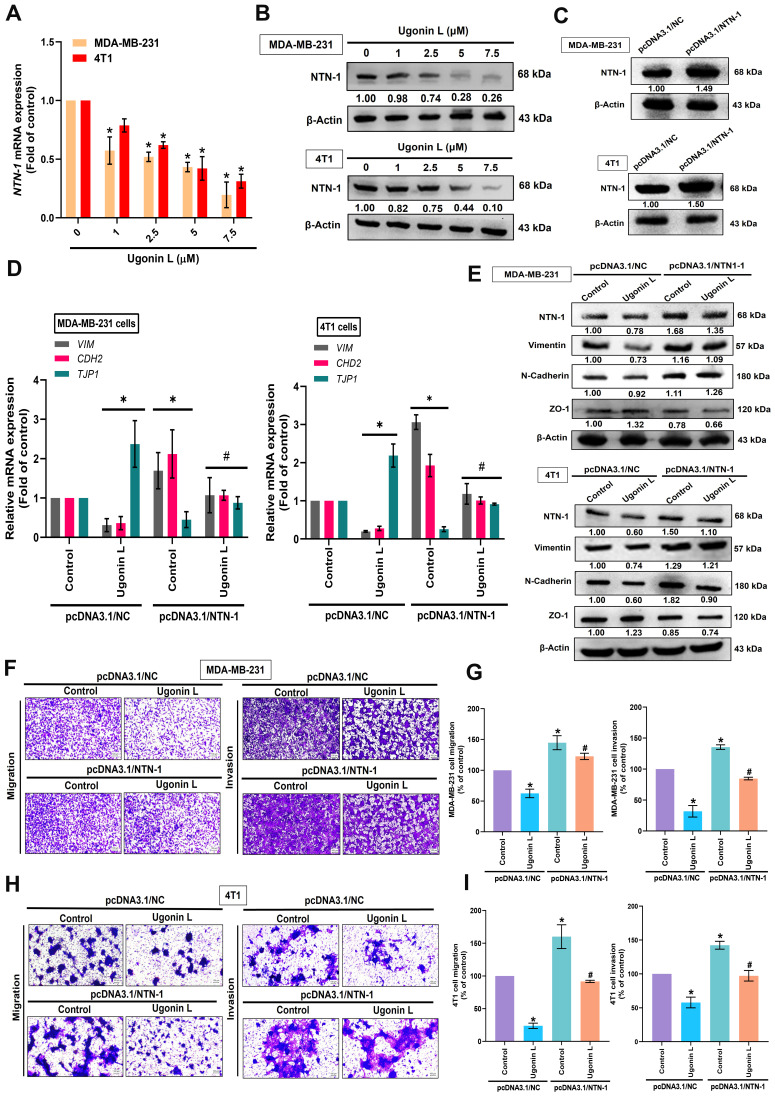
** Ugonin L inhibits breast cancer cell motility through reducing NTN-1 expression.** (A&B) Cells were treated with Ugonin L, the NTN-1 expression was examined by qPCR and Western blot. (C) Overexpression of *NTN-1* in MDA-MB-231 and 4T1 cells were confirmed by Western blotting following transfection with pcDNA3.1/NTN-1 compared with control vector (pcDNA3.1/NC). Cells were transfected with NTN-1 cDNA and treated with Ugonin L, the EMT markers (D&E), cell migraion and invasion (F-I) was examined. Results are presented as the mean ± SD of three independent biological experiments (n = 3). Scale bar = 100 μm. Protein band intensities were analyzed using ImageJ software. *p < 0.05 compared with the normal control group. #*p* < 0.05 compared with the Ugonin L-treated group.

To investigate the regulatory effect of Ugonin L on NTN-1, breast cancer cells were treated with Ugonin L, and both mRNA and protein expression levels of NTN-1 were significantly reduced (Figure 3A&B). Furthermore, NTN-1 overexpression markedly increased NTN-1 levels and reversed the inhibitory effects of Ugonin L on EMT marker expression, as well as cell migration and invasion (Figure 3C-I). Collectively, these results indicate that Ugonin L suppresses EMT and cell motility by downregulating NTN-1 expression in breast cancer cells.

### 3.3 Ugonin L blocks NTN-1-dependent EMT and motility in breast cancer cells by inhibiting the Wnt/β-catenin pathway

To examine detail molecular mechanism, we analysed the breast cancer bone metastasis GSE46141 dataset, the hallmark pathway enrichment analysis indicated that high NTN-1 expression was strongly linked with tissue invasion and metastasis signatures (Supplementary Figure 3A). Gene Ontology (GO) and KEGG pathway analyses of differentially expressed genes linked to breast cancer bone metastasis revealed that the Wnt signaling pathway was among the most markedly enriched pathways, alongside other processes related to breast cancer progression such as adherens junction regulation (Supplementary Figure 3B&C). Supporting these findings, Ingenuity Pathway Analysis (IPA) confirmed prominent activation of the Wnt/β-catenin signaling pathway in breast cancer bone metastatic samples (Supplementary Figure 3D). A detailed molecular interaction map revealed that GSK3β and β-catenin are key regulatory molecules within this signaling cascade (Supplementary Figure 3E).

**Figure 4:**
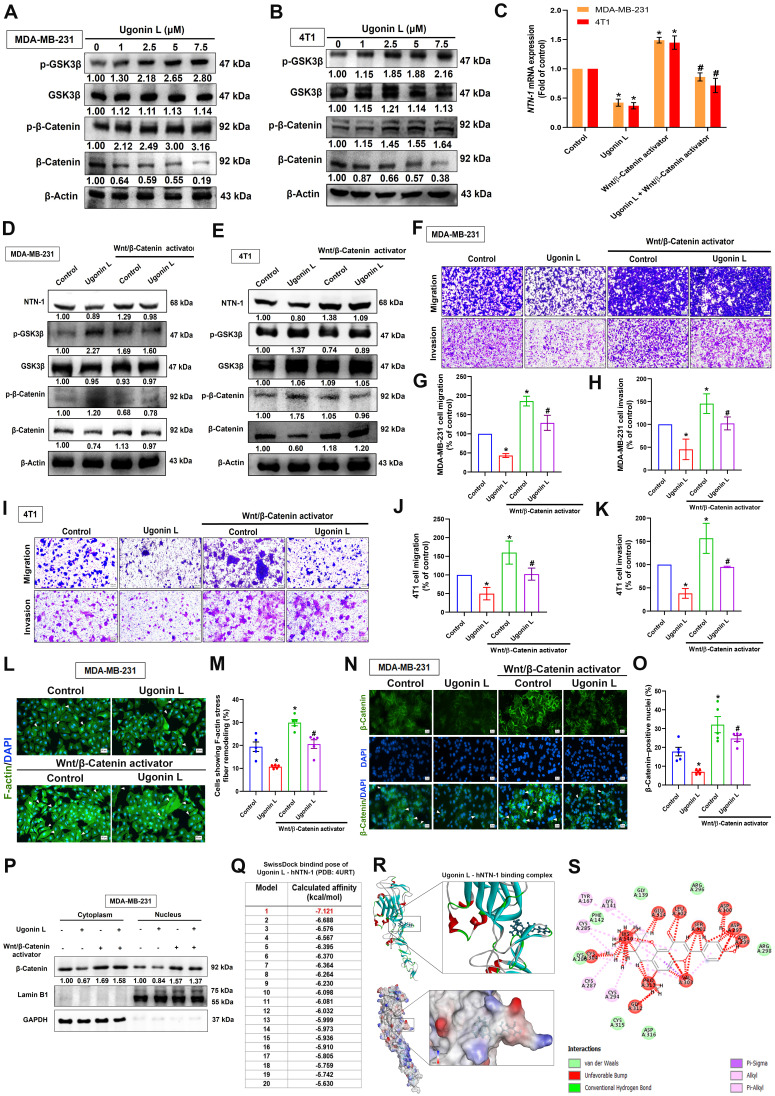
** Ugonin L inhibits breast cancer cell motility and EMT through modulation of Wnt/β-catenin signaling.** (A&B) Cells were treated with Ugonin L, the p-GSK3β and p-β-catenin was examined by Western blot. Results are presented as the mean ± SD of three independent biological experiments (n = 3). NTN-1 mRNA and protein expression in MDA-MB-231 and 4T1 cells treated with Ugonin L (7.5 μM), Wnt/β-catenin activator (5 nM), or their combination (C-E). Effects on cell migration and invasion were evaluated by transwell assays with corresponding quantification (F-K); Scale bar = 100 μm. F-actin remodeling and β-catenin nuclear translocation were analyzed by immunofluorescence staining and quantified (L-O); Scale bar = 20 μm. Cytoplasmic and nuclear β-catenin expression was further confirmed by fractionation assays (P). Molecular docking analysis revealed the binding interaction between Ugonin L and NTN-1 protein, docking poses were ranked based on predicted binding affinity (kcal/mol), and the top-ranked pose (lowest binding energy) was selected for further structural analysis (Q-S). White arrows indicate cells with F-actin remodeling or β-catenin nuclear translocation. Protein band intensities were analyzed using ImageJ software. Results are presented as the mean ± SD of three independent biological experiments (n = 3).^*^p < 0.05 compared with the control group. #*p* < 0.05 compared with the Ugonin L-treated group.

Western blot analysis showed that Ugonin L increased the phosphorylation of GSK3β and β-catenin while reducing total β-catenin expression, suggesting enhanced β-catenin turnover (Figure 4A&B). Treatment with the Wnt/β-catenin activator alone significantly increased NTN-1 mRNA and protein levels and was associated with reduced β-catenin phosphorylation and increased total β-catenin expression, consistent with β-catenin stabilization (Figure 4C-E). In contrast, co-treatment with the Wnt/β-catenin activator partially reversed the inhibitory effects of Ugonin L on NTN-1 mRNA and protein expression (Figure 4C-E) and was accompanied by restoration of cell migration and invasion (Figure 4F-K). In addition, immunofluorescence analysis showed that the Wnt/β-catenin activator attenuated the inhibitory effects of Ugonin L on F-actin stress fiber formation (Figure 4L&M). Furthermore, Wnt/β-catenin activator reversed the effect of Ugonin L on β-catenin nuclear localization, as shown by immunofluorescence staining and colocalization analysis with DAPI (Figure 4N&O). Subcellular fractionation analysis showed that Ugonin L reduced β-catenin levels in both the nuclear and cytoplasmic fractions, whereas co-treatment with the Wnt/β-catenin activator significantly restored these levels, particularly in the nuclear fraction (Figure 4P). These results further suggest that Wnt/β-catenin signaling acts upstream of NTN-1 to regulate cell motility, a process that is inhibited by Ugonin L.

To explore the potential direct interaction between Ugonin L and NTN-1, molecular docking analysis was performed. Among the 20 predicted binding poses, pose 1 exhibited the strongest binding affinity (-7.121 kcal/mol) (Figure 4Q). Ugonin L exhibited favorable predicted binding to human NTN-1, adopting a stable docking conformation with multiple putative interaction sites, including hydrogen bonds and hydrophobic contacts (Figure 4R&S). Based on the docking results, Asp297 and Ser301 were selected for site-directed mutagenesis, as both residues were predicted to form multiple hydrogen bonds with Ugonin L and were located near the center of the proposed binding pocket. Specifically, these two residues were mutated simultaneously to generate the NTN-1 double mutant D297A/S301A, in order to evaluate the potential contribution of this predicted binding region to the activity of Ugonin L. Transfection of the mutant NTN-1 overexpression plasmid resulted in NTN-1 expression levels comparable to those of the wild-type construct (Supplementary Figure 4A). Importantly, the anti-motility effects of Ugonin L were attenuated when the predicted NTN-1 binding site was mutated (Supplementary Figure 4B&C). These findings suggest that Ugonin L exerts its inhibitory function, at least in part, through direct interaction with NTN-1. Taken together, our data demonstrate that the suppression of NTN-1-mediated EMT and cell motility by Ugonin L is driven by its binding to NTN-1, alongside the modulation of the Wnt/β-catenin signaling pathway.

### 3.4. Ugonin L attenuates breast cancer-induced osteoclast differentiation, with NTN-1 as a potential mediator

**Figure 5:**
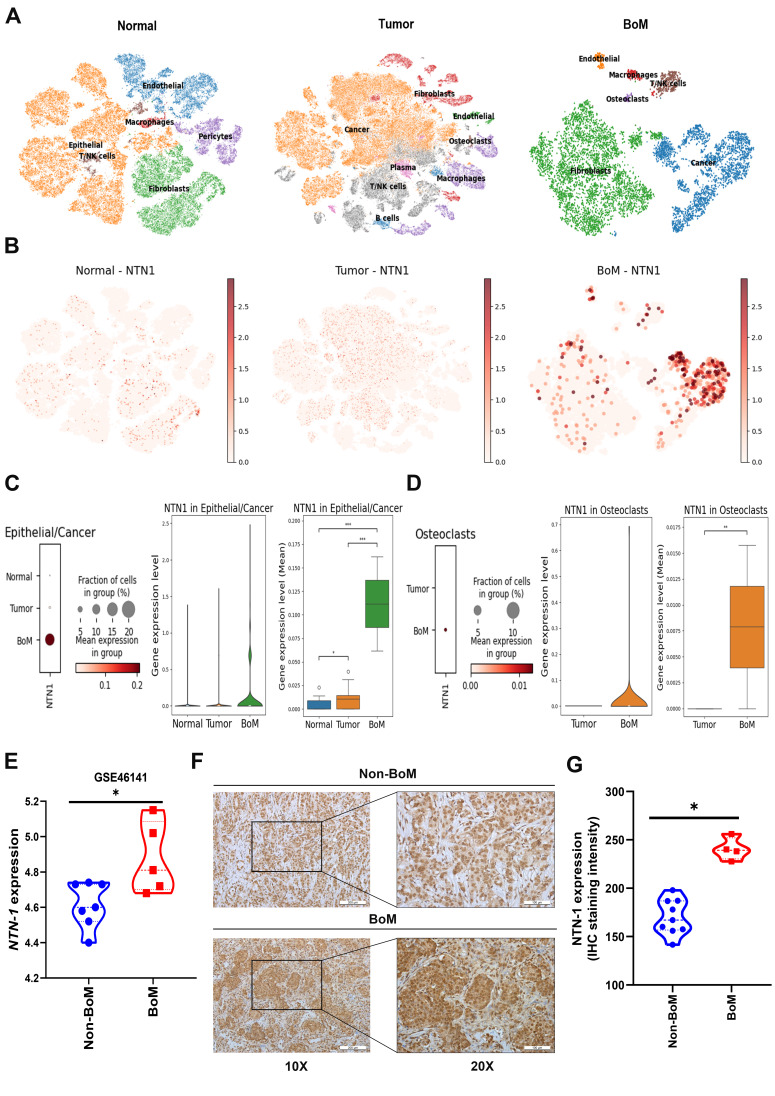
** NTN-1 is associated with breast cancer bone metastasis.** (A&B) UMAP embeddings of single-cell RNA-seq data from normal breast tissue, primary breast tumors, and bone-metastatic lesions, showing cell clustering and *NTN-1* gene expression levels across samples. (C&D) Single-cell RNA-seq analysis showing enriched *NTN-1* gene expression in malignant epithelial cells and osteoclasts in bone-metastatic lesions. (E) *NTN-1* gene expression in non-bone metastasis (Non-BoM; n = 7) and BoM samples (n = 5) from the Gene Expression Omnibus dataset GSE46141. (F) Representative IHC staining of NTN-1 protein in Non-BoM (n = 9) and BoM tissues (n = 4). The scale bars for 10X and 20X magnification correspond to 100 μm and 200 μm, respectively. (G) Quantification of NTN-1 IHC staining intensity. Data are presented as mean ± SD. *p < 0.05 compared with the control group.

To provide single-cell-level context for our experimental findings and explore the functional relevance of *NTN-1* in breast cancer bone metastasis, we analyzed publicly available scRNA-seq datasets derived from normal breast tissue, primary tumors, and bone metastatic lesions. The analysis revealed that *NTN-1* expression was enriched in bone metastatic (BoM) samples, with consistent expression observed across multiple cell populations, including malignant epithelial cells and osteoclasts (Figure 5A-D). Moreover, both osteoclast abundance and *NTN-1* expression within osteoclasts were markedly increased in patients with bone-metastatic breast cancer (Figure 5D). In addition, epithelial/tumor cell populations were validated by the expression of established markers, including Mucin 1 *(MUC1)*, Erb-B2 receptor tyrosine kinase 2* (ERBB2/HER2)*, tumor-associated calcium signal transducer 2 *(TACSTD2/EpCAM)*, keratin 5* (KRT5)*, and estrogen receptor 1* (ESR1)* 37, 38. Osteoclast populations were identified based on canonical markers, including acid phosphatase 5, tartrate-resistant *(ACP5/TRAP)*, cathepsin K* (CTSK)*, matrix metallopeptidase 9* (MMP9)*, Spi-1 proto-oncogene* (SPI1/PU.1)*, and *CD68* 39. These analyses were further used to characterize the tumor-bone microenvironment in BoM samples (Supplementary Figure 5), suggesting that *NTN-1* contributes to the interaction between tumor cells and osteoclasts within the metastatic niche.

**Figure 6:**
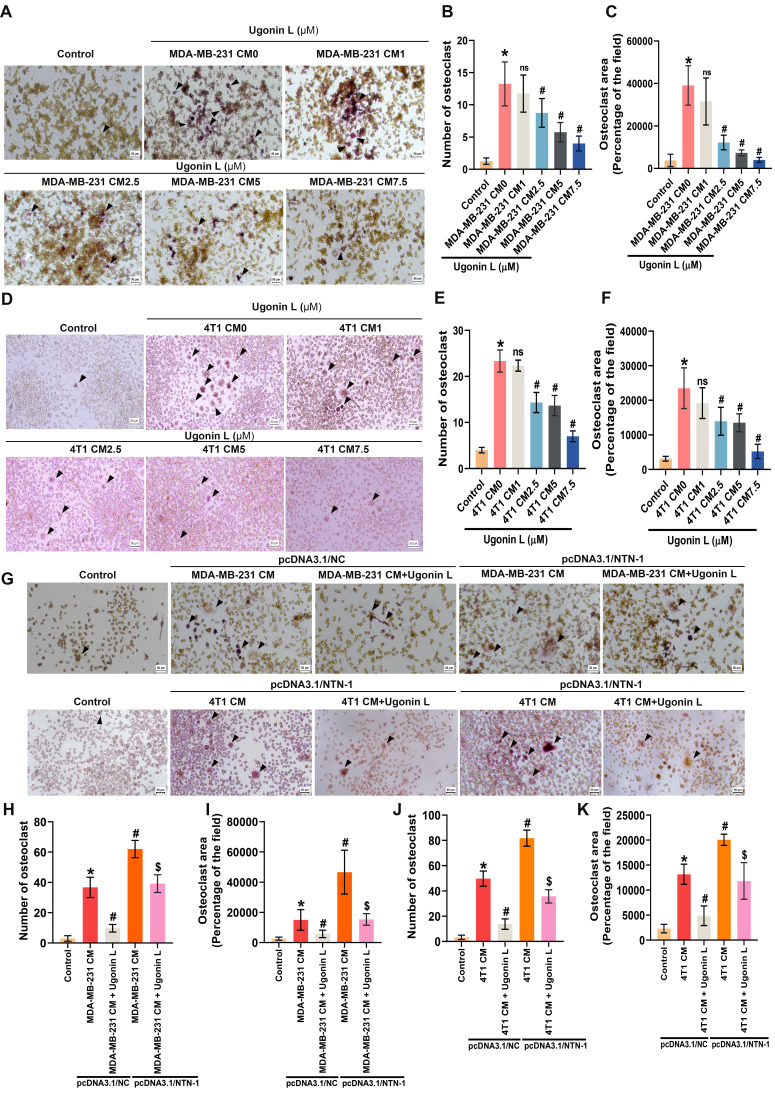
** Ugonin L inhibits breast cancer-induced osteoclast differentiation through NTN-1.** (A-F) RAW264.7 cells were cultured with CM derived from MDA-MB-231 or 4T1 cells-treated with Ugonin L for 5 days. Osteoclast area and number were assessed using TRAP assay. (G-K) MDA-MB-231 or 4T1 cells were transfected with *NTN-1* cDNA and treated with Ugonin L, the CM was collected and applied to RAW264.7 cells for 5 days. Osteoclast area and number were assessed using TRAP assay; black arrows indicate TRAP-positive osteoclasts. Results are presented as the mean ± SD of three independent biological experiments (n = 3). Scale bar = 100 μm. Osteoclast number and area were analyzed using ImageJ software. *p < 0.05 compared with the control group. #*p* < 0.05 compared with the conditioned medium-treated group. ^$^*p* < 0.05 compared with the Ugonin L-treated NC group.

Consistent with these findings, analysis of GEO datasets and our clinical cohort demonstrated that NTN-1 protein expression was significantly higher in primary breast cancer tissues from patients with bone metastasis compared with those without bone metastasis (Figure 5E-G). Furthermore, ELISA analysis showed that NTN-1 protein secretion in MDA-MB-231 and 4T1 cells were reduced in a dose-dependent manner following Ugonin L treatment, whereas *NTN-1* gene overexpression markedly increased its levels (Supplementary Figure 6A-C). qPCR analysis further revealed that *NTN-1* gene overexpression significantly upregulated osteoclast-related cytokines, including RANKL* (TNFSF11)*, *TNF-α*, *IL-1β*, and *IL-6*, in breast cancer cells (Supplementary Figure 6D&E), implying that NTN-1 serves as an upstream regulator of osteoclast-promoting cytokine networks in breast cancer cells. To further investigate the role of Ugonin L in cancer-induced osteoclast differentiation, RAW264.7 cells were cultured with conditioned medium (CM) derived from breast cancer cells. CM from breast cancer cells markedly promoted osteoclast formation, indicating that tumor-derived factors induce osteoclast differentiation (Figure 6A-F). In contrast, CM from Ugonin L-treated breast cancer cells significantly suppressed osteoclast formation (Figure 6A-F). Notably, *NTN-1* gene overexpression restored the osteoclast-promoting effect of CM, thereby counteracting the inhibitory effects of Ugonin L (Figure 6G-K). Collectively, these findings indicate that Ugonin L attenuates breast cancer-induced osteoclast differentiation, which is associated with the suppression of NTN-1.

### 3.5. Ugonin L reduces osteolytic bone metastasis *in vivo*

**Figure 7:**
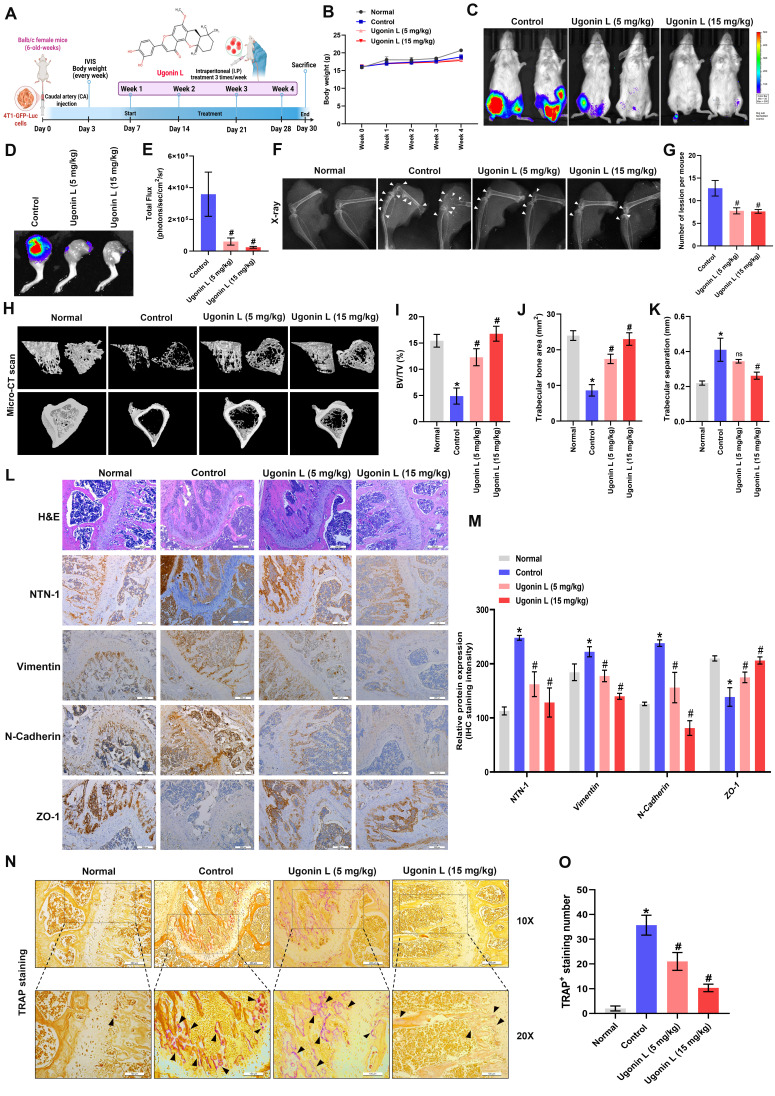
** Ugonin L suppresses osteolytic bone metastasis and NTN-1/EMT-associated signaling *in vivo*.** (A) Schematic illustration of the experimental design. 4T1-GFP-Luc breast cancer cells were injected via the caudal artery, and mice were treated intraperitoneally with Ugonin L (5 or 15 mg/kg) for 4 weeks. (B) Body weight of mice during the treatment period. (C-E) *Ex vivo* bioluminescence imaging of hind limbs with corresponding quantification. (F&G) Representative X-ray images of tibiae with corresponding quantification of bone resorption area. (H-K) Representative µCT reconstructions of femoral bones with corresponding quantitative analyses of bone parameters, including bone volume fraction (BV/TV), trabecular number (Tb.N) and trabecular separation. (L&M) Representative hematoxylin and eosin-stained and NTN-1 and EMT protein markers-immunostained tibial bone sections from normal, control, and Ugonin L-treated mice. Scale bar = 200 μm. (N&O) Representative TRAP-stained tibial bone sections from normal, control, and Ugonin L-treated mice. The scale bars for 10X and 20X magnification correspond to 100 μm and 200 μm, respectively. Results are presented as the mean ± SD of three independent biological experiments (n = 3). *p < 0.05 compared with the control group. #*p* < 0.05 compared with the Ugonin L-treated group.

To assess the therapeutic potential of Ugonin L *in vivo*, 4T1-GFP-Luc cells were injected into BALB/c mice via the caudal artery, followed by intraperitoneal administration of Ugonin L (5 or 15 mg/kg) (Figure 7A). After 4 weeks, 4T1-GFP-Luc cells had significantly metastasized to bone (Figure 7B-E). Ugonin L treatment reduced breast cancer metastasis to bone without affecting body weight, as examined by *in vivo* bioluminescence imaging (Fig. 7B-E). X-ray imaging showed suppressed tumor growth and bone erosion in Ugonin L-treated mice (Figure 7F&G). µCT analysis revealed that Ugonin L treatment markedly reversed extensive bone erosion, loss of bone volume/tissue volume ratio (BV/TV), trabecular number (Tb.N) and increase of trabecular separation (Figure 7H-K). H&E staining confirmed the osteolytic nature of bone lesions formed by 4T1-GFP-Luc cells and their inhibition by Ugonin L (Figure 7L). IHC staining showed that NTN-1 and EMT markers expression in tumor tissue was markedly reversed in Ugonin L-treated groups (Figure 7L&M). Ugonin L also decreased osteoclast number, as shown by TRAP staining (Figure 7N&O). These results indicate that Ugonin L suppresses breast cancer-induced osteolytic bone metastasis by downregulating NTN-1 and limiting osteoclast activation.

## 4. Discussion

Patient survival and quality of life are significantly impacted by osteolytic bone metastases linked to breast cancer. Bone-derived growth factors maintain a vicious cycle that promotes tumor survival in bone, resulting in bone degradation, whereas tumor-derived factors such RANKL, MMP7, and IL-6 disturb osteoclast-osteoblast balance 40, 41. The use of existing therapies, such as denosumab and bisphosphonates, is restricted by side effects such hypercalcemia and osteonecrosis 42, 43. Finding new drugs that interfere with tumor-bone interactions can therefore be a crucial therapeutic objective. As a pharmacological agent for anticancer medications, traditional Chinese medicine has been extensively researched 44, 45. In the last few years, several traditional Chinese herbal medicine components have demonstrated antitumor effects 46-48. Ugonins, isolated from *H. zeylanica*, showed potent biological and pharmacological features, such as anti-inflammatory 21, 49, 50, immunomodulatory 23, 51, and anti-cancer activities 22, 26. However, its function in osteolytic bone metastasis has not been previously explored. Here, we show that Ugonin L inhibits breast cancer EMT and cell motility as well as breast cancer-facilitated osteoclast formation by inhibiting NTN-1 synthesis. Importantly, Ugonin L also blocks breast cancer metastasis to bone and suppresses osteoclast formation *in vivo*. Thus, Ugonin L represents a novel therapeutic agent for treating breast cancer-induced osteolytic bone metastasis.

A secreted glycoprotein called NTN-1 is essential for angiogenesis, cell survival, and neural navigation 52. Additionally, NTN-1 has been linked to a number of illnesses, such as cancer, inflammatory diseases, polycystic kidney disease, and cardiovascular disease 53, 54. Numerous malignancies have been shown to have up-regulated NTN-1, and this up-regulation has been demonstrated to comprise a selective mechanism that prevents apoptosis 52. Here, we identified NTN-1 as a key mediator of Ugonin L-induced anti-metastatic effects. Clinical data further demonstrated that high NTN-1 expression is linked with breast cancer progression, bone metastasis, and poorer patient survival.

Ugonin L treatment reduced NTN-1 expression in breast cancer cells, while overexpression of NTN-1 antagonized the Ugonin L-induced reduction of breast cancer cell EMT and motility. Consistently, the *in vivo* results also showed that Ugonin L suppressed NTN-1 expression and breast cancer bone metastasis. Together, these findings indicate that NTN-1 is a key mediator of Ugonin L-induced suppression of breast cancer bone metastasis. We also employed a computational model to predict whether Ugonin L directly interacts with NTN-1. Molecular docking analysis revealed a potential interaction between Ugonin L and NTN-1, with a docking energy of -7.121 kcal/mol. In addition, docking analysis provides only predictive evidence of Ugonin L-NTN-1 interaction. Although mutagenesis supports functional relevance, further studies are required to confirm direct binding using biochemical assays. These results suggest that Ugonin L regulates NTN-1 expression not only through cellular signaling mechanisms but also via direct binding to NTN-1.

Metastasis is a multi-step biological process that accounts for most breast cancer-related fatalities rather than the primary tumor. Notably, EMT plays a critical role in the migration and metastasis of cancer as well as in the genesis of malignancies. EMT is thought to be activated by cancer cells, enabling them to detach from the primary tumor and enter blood vessels 55. During EMT, epithelial cells acquire a mesenchymal character with enhanced motility, losing their adherents junctions and apical-basal polarity. EMT inhibition is therefore a desirable therapeutic approach. The results of this study demonstrated that Ugonin L treatment in breast cancer cells inhibits EMT by upregulating epithelial cell markers and downregulating mesenchymal cell markers. Targeting NTN-1 has been indicated to control cancer EMT and metastasis 56. Interestingly, ectopic overexpression of NTN-1 cDNA completely abolished the Ugonin L-mediated inhibition of EMT marker changes in breast cancer cells. Consistently, our *in vivo* mouse model revealed that Ugonin L similarly suppressed NTN-1 expression and reversed EMT marker alterations. Collectively, these findings establish NTN-1 as a major downstream mediator of Ugonin L-induced suppression of EMT and motility in breast cancer.

Osteoclast production is stimulated by tumor-derived substances from breast cancer cells via both RANKL-dependent and -independent pathways 57. Specifically, CM from MDA-MB-231 cells promotes osteoclast development and initiates a vicious cycle of bone loss that is reinforced by RANKL-independent signaling mediated by IL-8 58, 59. CM collected from NTN-1-overexpressing breast cancer cells reversed the inhibitory effect of Ugonin L, restoring osteoclast formation. In contrast, Ugonin L treatment effectively suppressed NTN-1 expression, osteoclast numbers, and breast cancer-induced osteolytic bone metastasis *in vivo*. To further support this mechanism, NTN-1 protein levels were quantified by ELISA (Supplementary Figure 6A-C), and the expression of key osteoclastogenic cytokines, including RANKL, TNF-α, IL-6, and IL-1β, was assessed by qPCR under NTN-1 overexpression conditions with or without Ugonin L treatment (Supplementary Figure 6D&E). NTN-1 overexpression was associated with increased expression of these osteoclast-related factors, implying that NTN-1 acts as an upstream regulator of osteoclast-promoting cytokine networks in breast cancer cells. Together, these findings suggest that NTN-1 contributes to osteoclastogenesis, at least in part, by enhancing a pro-osteoclastogenic tumor secretome.

Although our data support the involvement of the Wnt/β-catenin/NTN-1 axis in the inhibitory effects of Ugonin L on EMT and metastatic behaviour, we acknowledge that Ugonin L has also been reported to modulate additional signaling pathways, including MAPK and NF-κB 25. These pathways are known to interact with Wnt/β-catenin signaling and to converge on the regulation of EMT, cell migration, and tumor progression 60, 61. Therefore, it is possible that the anti-cancer effects of Ugonin L involve coordinated modulation of multiple pathways, with Wnt/NTN-1 signaling representing one important, but not exclusive, mechanism. Notably, NTN-1 overexpression partially reversed the inhibitory effects of Ugonin L, supporting a functional role for NTN-1 in this regulatory network. In addition, mutation of the predicted binding site attenuated the inhibitory effects of Ugonin L on cell motility, further suggesting that interaction with NTN-1 contributes to its functional activity. Overall, our findings support a functional association between Ugonin L and the inhibition of EMT and cell motility, potentially through interaction with NTN-1 and modulation of Wnt/β-catenin signaling.

Although our bioinformatics analyses indicate that elevated NTN-1 expression is associated with breast cancer progression, bone metastasis, and poor prognosis, these findings remain correlative rather than causal. Public datasets, such as TCGA and GEO, can be influenced by sample heterogeneity and clinical confounding factors. To strengthen the robustness of our results, we validated NTN-1 expression across multiple independent cohorts and clinical samples, consistently observing higher expression in metastatic and aggressive tumors. Importantly, our *in vitro* and *in vivo* experiments further demonstrate that NTN-1 promotes EMT, osteoclastogenesis, and bone metastatic phenotypes. Collectively, these findings support a functional role for NTN-1, although further prospective studies are needed to confirm its independent prognostic value and definitive causal contribution.

In our study, both MDA-MB-231 and 4T1 TNBC cell lines exhibited reduced migratory and invasive capacities following Ugonin L treatment. To further examine whether Ugonin L also affects non-TNBC breast cancer cells, we performed migration and invasion assays using MCF-7 and T-47D cells. The results showed that Ugonin L suppressed the motility of these non-TNBC cells without significant cytotoxicity (Supplementary Figure 1). Given that clinical cohort analysis revealed significantly higher *NTN-1* expression in TNBC than in non-TNBC subtypes (Figure 2D), these findings suggest that the inhibitory effects of Ugonin L in non-TNBC cells involve additional mechanisms beyond NTN-1. We found that Ugonin L suppresses breast cancer cell motility and EMT, which is associated with the inhibition of Wnt/β-catenin signaling and a potential interaction with NTN-1. Furthermore, Ugonin L attenuated NTN-1-associated, tumor cell-induced osteoclast formation, accompanied by the reduced expression of pro-osteoclastogenic cytokines, including *RANKL*, *TNF-α*, *IL-6*, and *IL-1β*; notably, *NTN-1* cDNA overexpression partially reversed these inhibitory effects. These findings suggest a potential link between EMT regulation and the tumor secretome. Accordingly, modulation of this pathway reduces osteoclast-stimulating signals, with NTN-1 potentially acting as a molecular mediator between tumor progression and osteoclastogenesis.

**Figure 8:**
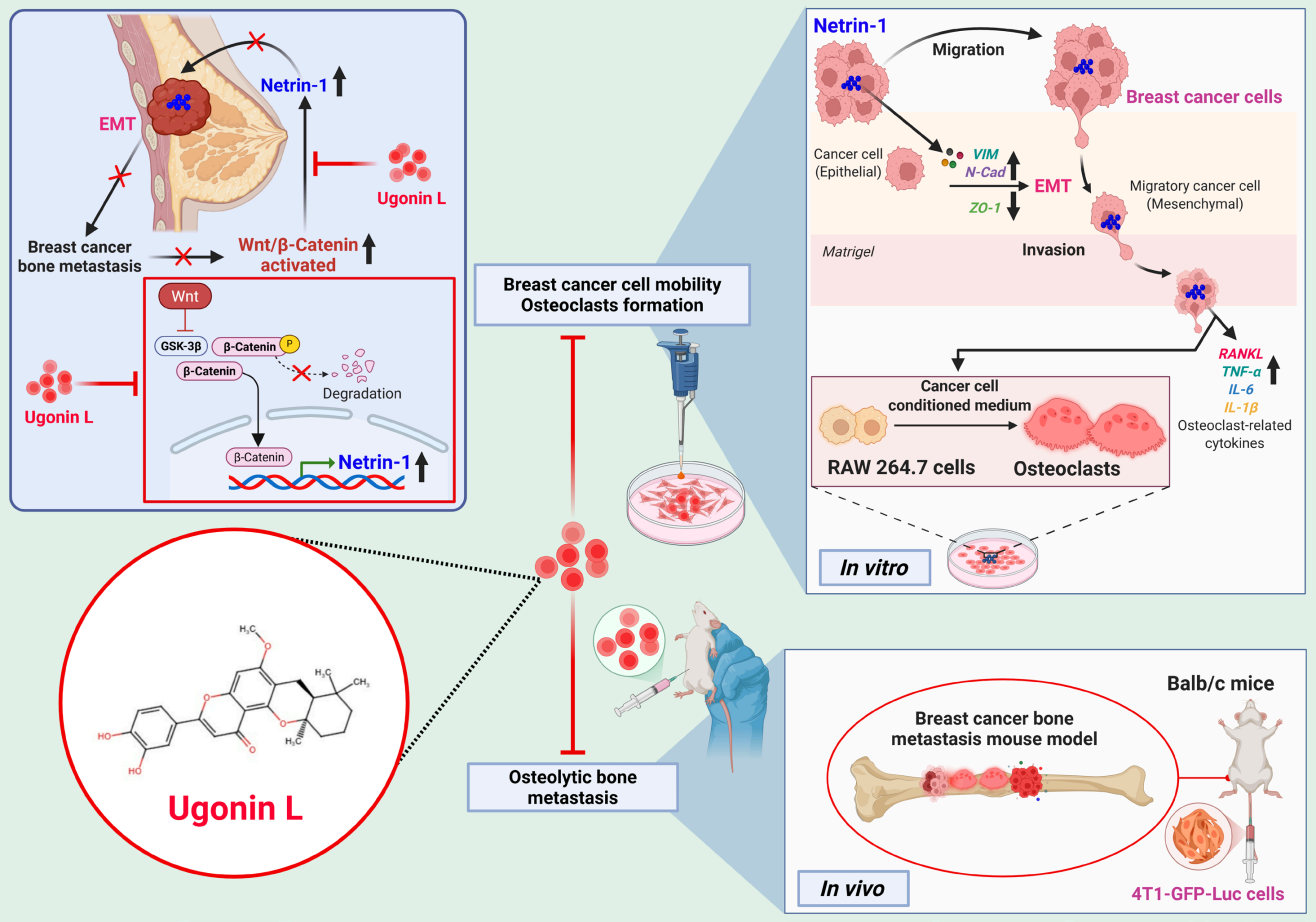
** Schematic representation illustrates that Ugonin L inhibits breast cancer osteolytic bone metastasis.** Ugonin L inhibits NTN-1-dependent EMT and motility in breast cancer through inhibiting the Wnt/β-catenin pathway. Ugonin L also attenuates breast cancer-induced osteoclast formation, resulting in inhibition of breast cancer osteolytic bone metastasis.

In summary, Ugonin L inhibits breast cancer cell EMT, migration and invasion. NTN-1 mediates the effects of Ugonin L through the Wnt/β-catenin pathway. Ugonin L also attenuates breast cancer-induced osteoclast formation by inhibiting NTN-1 expression (Figure 8). Molecular docking results further indicate a potential interaction between Ugonin L and NTN-1 protein. Importantly, Ugonin L blocks breast cancer-induced osteolytic bone metastasis *in vivo*. These findings highlight Ugonin L as a promising therapeutic candidate for the remedy of breast cancer osteolytic bone metastasis.

## Supplementary Material

Supplementary figures, methods and tables.
