# Supplementary material for: Targeting Netrin-1 Mediates the Suppression of Osteolytic Bone Metastasis in Breast Cancer by Ugonin L
Source: Int J Biol Sci. 2026 Jun 10;22(12):6298–315. doi: 10.7150/ijbs.128973 (PMC13411717; doi:10.7150/ijbs.128973)
Supplement: Supplementary file 1 — Supplementary figures, methods and tables. [file ijbsv22p6298s1.pdf]

Targeting Netrin-1 Mediates the Suppression of Osteolytic Bone Metastasis in Breast Cancer by Ugonin L

Trung-Loc Ho[1,2#], Wei-Cheng Chen[3,4#], Chun-Lin Liu[5], Kuan-Ying Lai[6], Chih-Chuang Liaw[6,7], Lan-Yun Chang[8], Chih-Hao Lu[8,9], Hsiao-Chi Tsai[10], Yi-Chin Fong[11,12,13], Jeng-Hung Guo[5], Tzu-Min Huang[14], Chih-Ying Wu[15,16*], and Chih-Hsin Tang[1,17,18*]

[1]Department of Pharmacology, School of Medicine, China Medical University, Taichung, Taiwan.

[2]CirTech Institute, HUTECH University, Ho Chi Minh City, Vietnam.

[3]Department of Medicine, MacKay Medical University, New Taipei, Taiwan.

[4]Division of Sports Medicine & Surgery, Department of Orthopedic Surgery, MacKay Memorial Hospital, Taipei, Taiwan.

[5]Department of Neurosurgery, China Medical University Hospital, Taichung, Taiwan.

[6]Department of Marine Biotechnology and Resources, National Sun Yat-sen University, Kaohsiung, Taiwan.

[7]Graduate Institute of Natural Products, Kaohsiung Medical University, Kaohsiung, Taiwan

[8]Institute of Bioinformatics and Systems Biology, National Yang Ming Chiao Tung University, Hsinchu, Taiwan.

[9]Center for Intelligent Drug Systems and Smart Bio-Devices (IDS^{2}B), National Yang Ming Chiao Tung University, Hsinchu, Taiwan.

[10]Department of Medicine Research, China Medical University Beigang Hospital, Yunlin, Taiwan.

[11]Department of Sports Medicine, College of Health Care, China Medical University, Taichung, Taiwan.

[12]Department of Orthopedic Surgery, China Medical University Hospital, Taichung, Taiwan.

[13]Department of Orthopedic Surgery, China Medical University Beigang Hospital, Yunlin, Taiwan.

[14]Department of Chest Surgery, China Medical University Hospital, Taichung, Taiwan.

[15]School of Chinese Medicine, China Medical University, Taichung, Taiwan.

[16]Department of Neurosurgery, China Medical University Hsinchu Hospital, Hsinchu, Taiwan.
[17]Chinese Medicine Research Center, China Medical University, Taichung, Taiwan.
[18]Department of Medical Laboratory Science and Biotechnology, College of Medical and Health Science, Asia University, Taichung, Taiwan.

#These authors contributed equally to this work.

[*]**Corresponding authors:**

Chih-Hsin Tang, PhD; E-mail: chtang@mail.cmu.edu.tw

Chih-Ying Wu, MD; PhD; E-mail: 014345@tool.caaumed.org.tw

Supplementary Data

Supplementary results

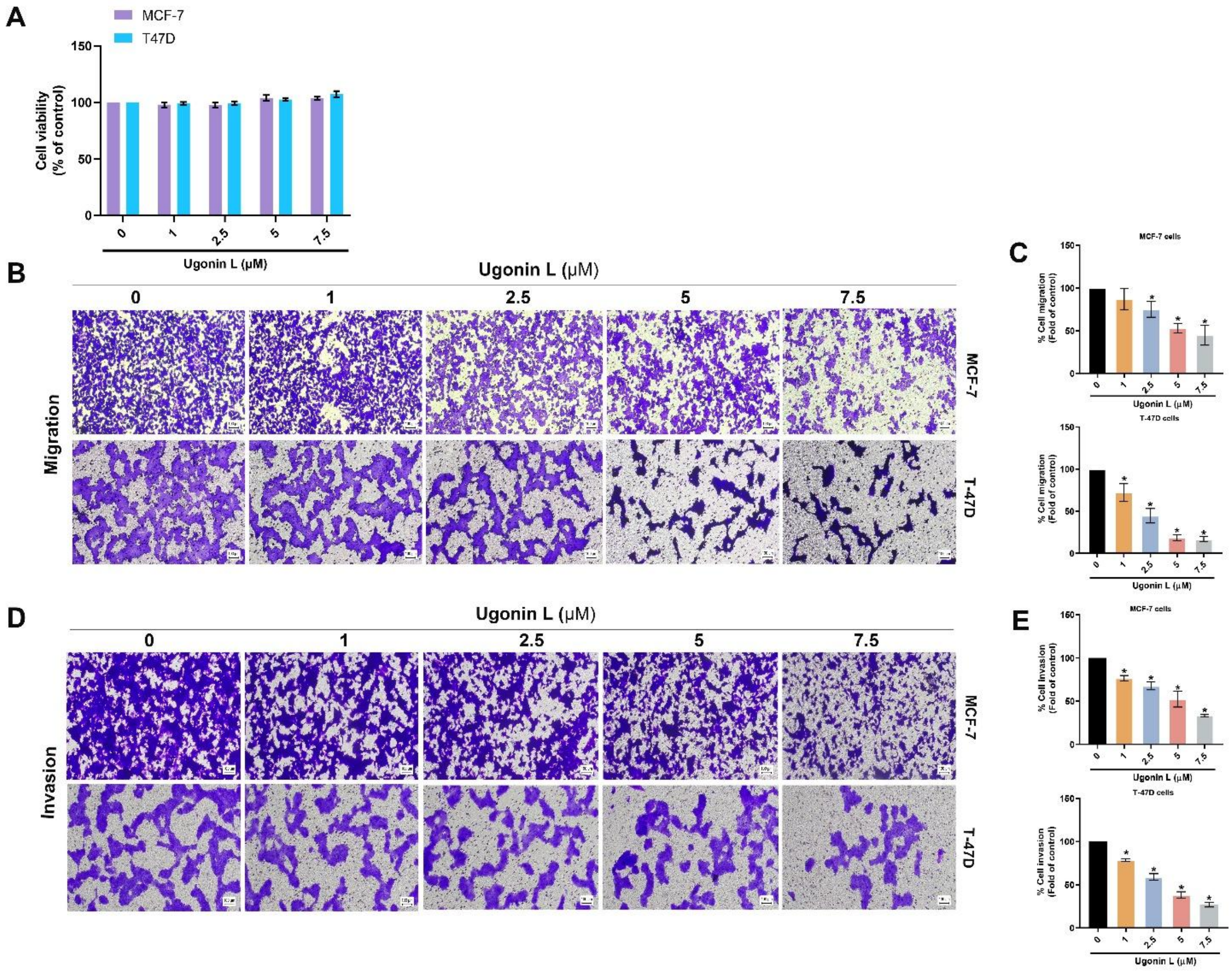

Supplementary Figure 1. Effects of Ugonin L on cell viability, migration, and invasion in human non-TNBC cells. Cell viability of MCF-7 and T-47D cells treated with Ugonin L at the indicated concentrations was assessed by MTT assay (A). Representative images of transwell migration (B) and invasion assays (D) in MCF-7 and T-47D cells following Ugonin L treatment. Quantitative analysis of migrated (C) and invaded cells (E) is shown. Data are presented as mean ± SD. $^{*}p < 0.05$ compared with the control group.

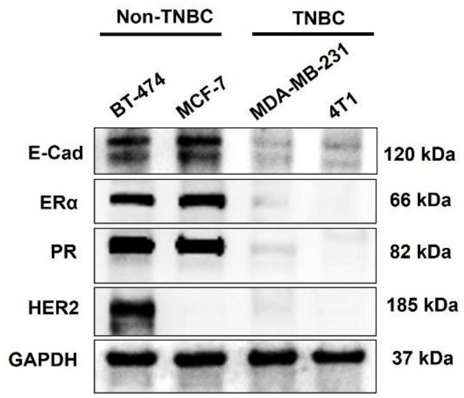

Supplementary Figure 2. Characterization of receptor status and E-cadherin expression in breast cancer cell lines. Western blot analysis was performed to examine E-cadherin (E-Cad), estrogen receptor alpha (ERα), progesterone receptor (PR), and human epidermal growth factor receptor 2 (HER2) protein expression in non-TNBC breast cancer cells (BT-474 and MCF-7) and TNBC-like breast cancer cells (MDA-MB-231 and 4T1). Equal amounts of total protein lysate (30 µg) were loaded per lane, and GAPDH was used as the loading control.

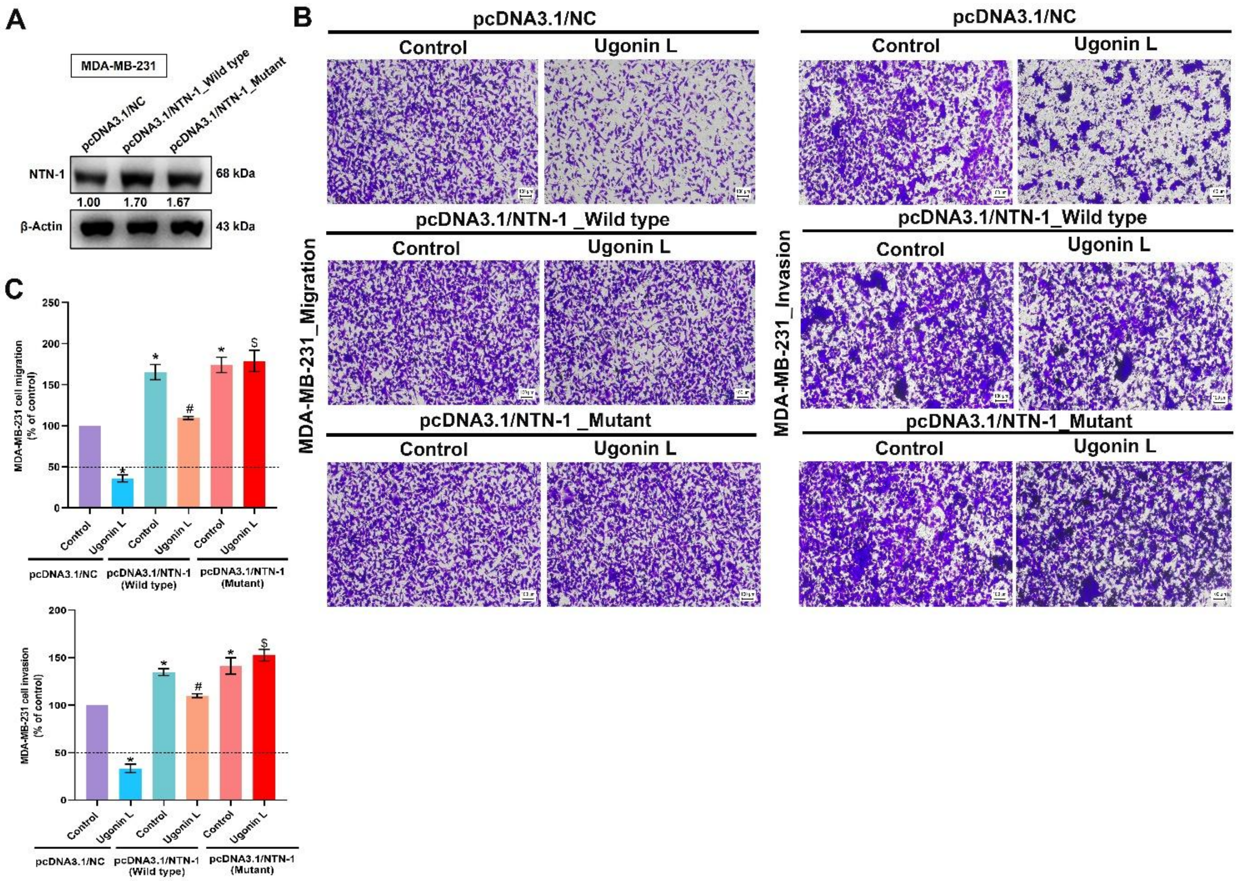

Supplementary Figure 3. Effects of NTN-1 mutation on Ugonin L–mediated inhibition of migration and invasion in breast cancer cells. NTN-1 protein expression in MDA-MB-231 cells transfected with control vector (pcDNA3.1/NC), NTN-1 wild-type, or NTN-1 mutant plasmids was analyzed by western blotting (A). Representative images of transwell migration and invasion assays in MDA-MB-231 cells under the indicated transfection conditions with or without Ugonin L treatment (B). Quantitative analysis of migrated and invaded cells is shown in (C). Data are presented as mean ± SD. $^{*}p < 0.05$ compared with the control group; $^{\#}p < 0.05$ compared with the pcDNA3.1/NC group; $^{\$}p < 0.05$ compared with the Ugonin L-treated pcDNA3.1/NTN-1 (wild-type) group.

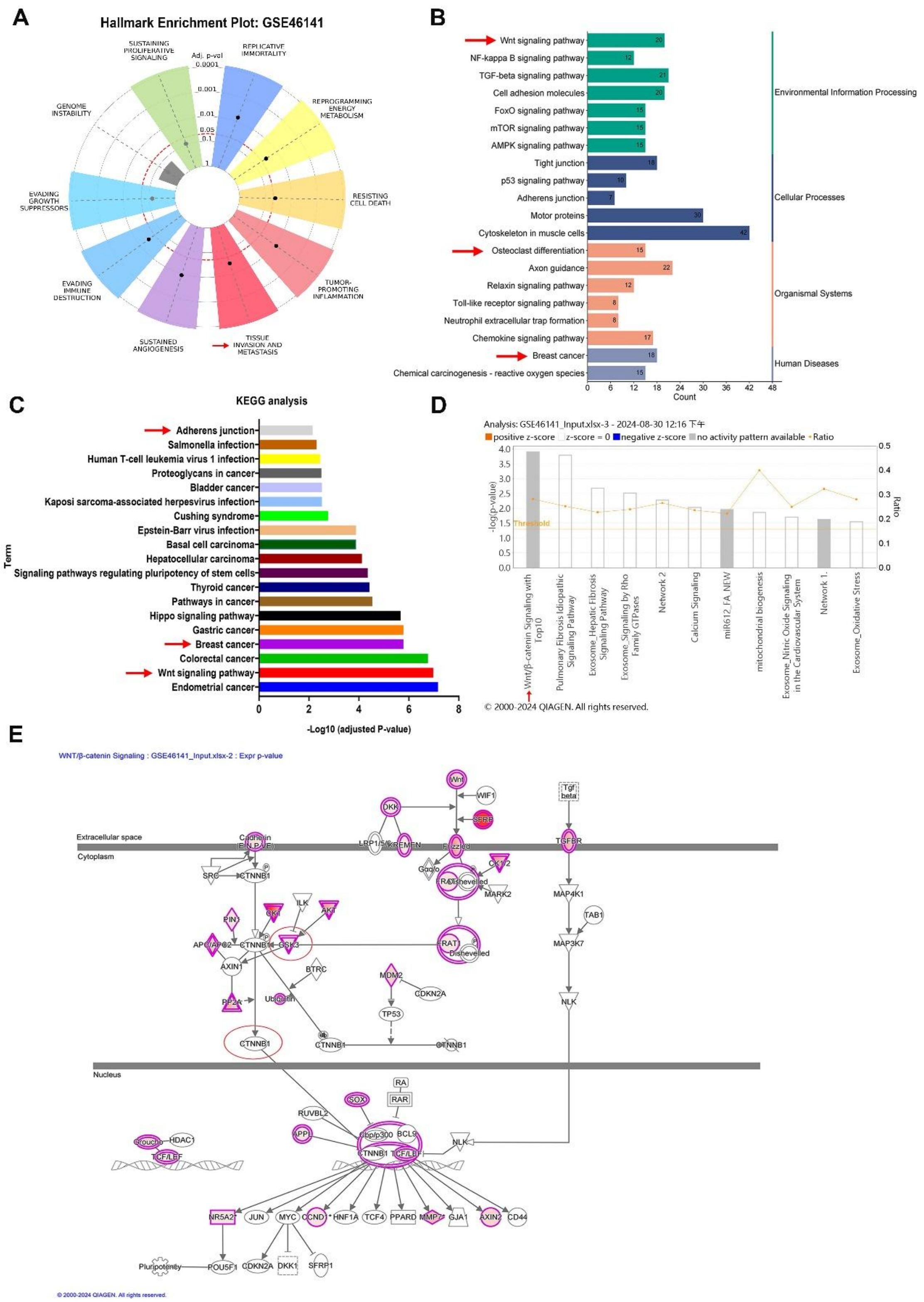

Supplementary Figure 4. Bioinformatic analyses reveal enrichment of invasion, metastasis, and Wnt/β-Catenin signaling pathways in breast cancer bone metastasis. (A) Hallmark pathway

enrichment analysis of the GSE46141 breast cancer bone metastasis dataset showing that high *NTN-1* expression strongly correlates with tissue invasion and metastasis signatures. (B&C) Gene Ontology (GO) and KEGG pathway analyses of differentially expressed genes in bone-metastatic breast cancer, highlighting significant enrichment of the Wnt signaling pathway and related processes. (D&E) Ingenuity Pathway Analysis (IPA) confirming robust activation of the canonical Wnt/β-catenin signaling pathway in bone-metastatic samples.

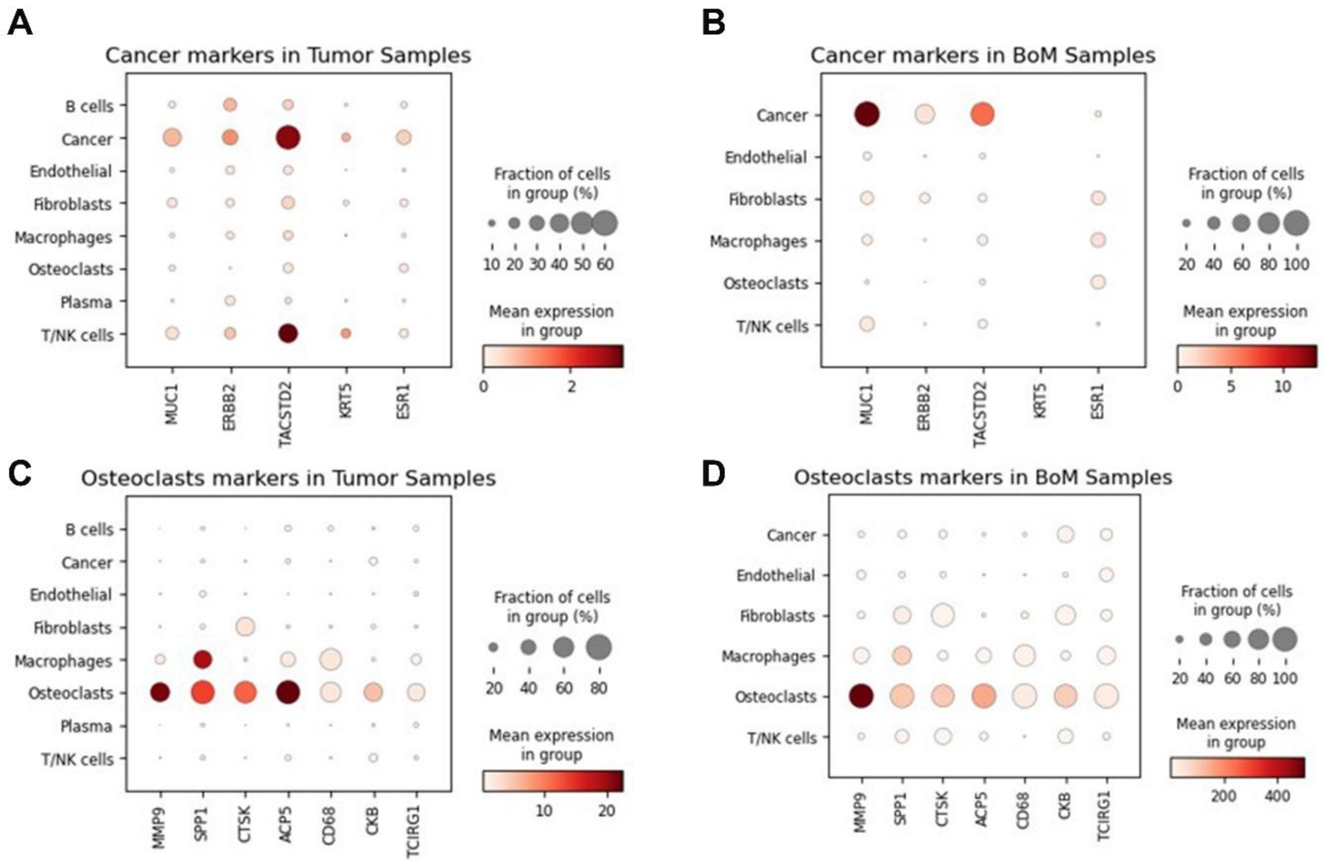

Supplementary Figure 5. Expression of cancer and osteoclast markers in tumor and bone metastasis samples. Dot plot analysis showing the expression of cancer-associated markers (*MUC1*, *ERBB2*, *TACSTD2*, *KRT5*, and *ESR1*) across different cell populations in tumor (A) and bone metastasis (BoM) samples (B). Expression of osteoclast-related markers (*MMP9*, *SPP1*, *CTSK*, *ACP5*, *CD68*, *CKB*, and *TCIRG1*) in tumor (C) and BoM samples (D). Dot size represents the fraction of cells in each group, and color intensity indicates the mean expression level.

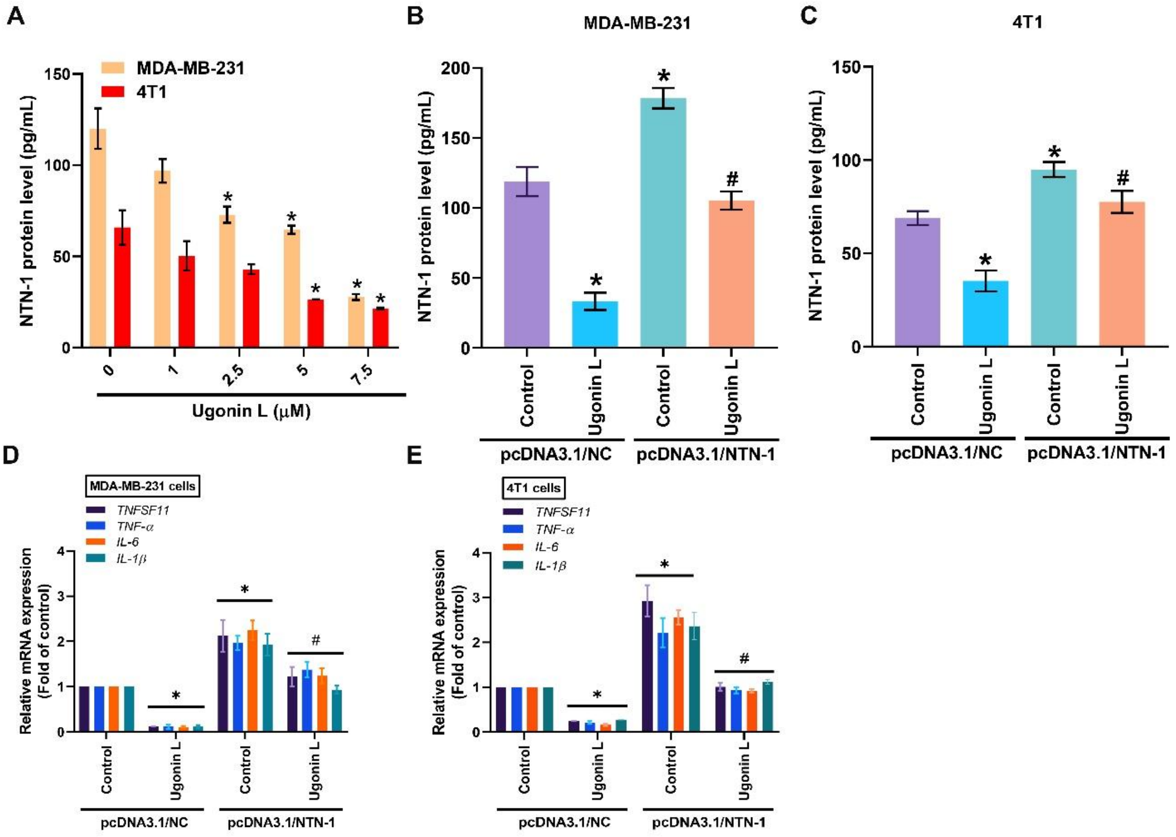

Supplementary Figure 6. Ugonin L reduces NTN-1 secretion and osteoclastogenic factor expression in breast cancer cells. NTN-1 protein levels in conditioned media from MDA-MB-231 and 4T1 cells treated with Ugonin L at the indicated concentrations were measured by ELISA (A). Effects of *NTN-1* cDNA overexpression on NTN-1 protein secretion in MDA-MB-231 (B) and 4T1 cells (C) with or without Ugonin L treatment. Relative mRNA expression levels of osteoclast-related factors, including *TNFSF11*, *TNF-α, IL-1β*, and *IL-6*, in MDA-MB-231 (D) and 4T1 cells (E) under the indicated conditions. Data are presented as mean ± SD. $p < 0.05$ compared with the control group; #$p < 0.05$ compared with the pcDNA3.1/NC group.

Supplementary method

MTT assay

Cell viability was determined using the MTT assay. Briefly, cells were seeded in 96-well plates at a density of 5 × 10^4 cells/well and allowed to adhere overnight. After the indicated treatments, 50 µL of MTT solution (5 mg/mL in PBS; Sigma-Aldrich, St. Louis, MO, USA) was added to each well and incubated for 4 h at 37 °C. The resulting formazan crystals were dissolved in 100 µL of dimethyl sulfoxide (DMSO; Sigma-Aldrich, USA), and absorbance was measured at 570 nm using a microplate reader (BioTek, Winooski, VT, USA). Cell viability was expressed as a percentage relative to untreated control cells.

Cell transfection assay

Breast cancer cells were seeded and cultured to approximately 70–80% confluence before transfection. Cells were transfected with *NTN-1* gene overexpression plasmid or the corresponding control vector using Lipofectamine 2000 (Invitrogen, Carlsbad, CA, USA) according to the manufacturer's protocol. Briefly, plasmid DNA and Lipofectamine 2000 were diluted separately in Opti-MEM medium, incubated for 5 min, and then combined for 20 min at room temperature to form transfection complexes. The complexes were added to the cells and incubated for 4–6 h, followed by replacement with fresh complete medium. After 24–48 h, transfected cells were harvested for further experiments.

Boyden chamber migration and invasion assay

Cell migration and invasion were assessed using Boyden chamber assays (8-µm pore size; Corning, NY, USA). For the migration assay, cells (1 × 10^4 cells/well) suspended in serum-free medium were seeded into the upper chamber, while the lower chamber was filled with medium containing 10% FBS as a chemoattractant. For the invasion assay, the upper chamber membrane was precoated with Matrigel (BD Biosciences, San Jose, CA, USA) and allowed to solidify before cell seeding [1]. After 24 h at 37 °C, non-migrated/non-invaded cells on the upper surface of the

membrane were removed with a cotton swab, and cells that had migrated or invaded to the underside were fixed with 4% paraformaldehyde, stained with 0.1% crystal violet, and counted under a light microscope in five randomly selected fields. Results were expressed as the average number of migrated or invaded cells per field.

Real-time quantitative PCR

Total RNA will be isolated from breast cancer cells using TRIzol™ reagent (MDBio, Taipei, Taiwan). qPCR will be conducted as previously reported. RNA concentration will be determined with a NanoVue Plus™ spectrophotometer (Biochrom Ltd., Cambridge, UK). Subsequently, 1 μg of total RNA will be reverse transcribed into complementary DNA (cDNA) using the MMLV reverse transcription system (Invitrogen, Carlsbad, CA, USA) and combined with Fast SYBR® Green Mix. Gene expression levels will be measured on the StepOnePlus™ Real-Time PCR System (Applied Biosystems, Foster City, CA, USA), with glyceraldehyde 3-phosphate dehydrogenase (GAPDH) serving as the internal control. The detailed primer sequences as provided in the Supplementary Table 1.

Western blotting assay

Cells were lysed in RIPA buffer (Thermo Fisher Scientific, Waltham, MA, USA) supplemented with protease and phosphatase inhibitor cocktails (Roche, Basel, Switzerland). Protein concentrations were determined using the BCA protein assay kit (Pierce, Rockford, IL, USA). Equal amounts of protein (30 μg) were separated by SDS-PAGE and transferred onto polyvinylidene difluoride (PVDF) membranes (Millipore, Billerica, MA, USA) [2]. Membranes were blocked with 5% non-fat milk in TBST (20 mM Tris-HCl, 150 mM NaCl, 0.1% Tween-20, pH 7.4) for 1 h at room temperature and then incubated overnight at 4 °C with primary antibodies against the indicated proteins (dilution 1:1,000). After washing, membranes were incubated with HRP-conjugated secondary antibodies (1:5,000; Jackson ImmunoResearch, West Grove, PA, USA) for 1 h at room temperature. Protein bands were visualized using an enhanced chemiluminescence

(ECL) detection kit (GE Healthcare, Little Chalfont, UK) and captured with the iBright FL1500 Imaging System (Thermo Fisher Scientific, USA). Protein band intensities were quantified using ImageJ software (version 1.49; National Institutes of Health, Bethesda, MD, USA).

Nuclear and cytoplasmic fractionation

Nuclear and cytoplasmic protein fractions were prepared using a nuclear and cytoplasmic extraction kit (G-Biosciences, Cat. No. 786-182) according to the manufacturer's instructions. The expression of β-catenin in nuclear and cytoplasmic fractions was analyzed by western blotting as described above. Membranes were incubated with mouse anti-β-catenin antibody (1:1,000; Santa Cruz Biotechnology, Dallas, TX, USA), mouse anti-GAPDH antibody (1:1,000; Santa Cruz Biotechnology, Dallas, TX, USA), and mouse anti-Lamin B1 monoclonal antibody (1:1,000; Santa Cruz Biotechnology, Dallas, TX, USA) overnight at 4 °C. After washing, membranes were incubated with HRP-conjugated goat anti-mouse IgG secondary antibody (1:3,000; Millipore, MA, USA; Cat. No. AP307P) for 1 h at room temperature. GAPDH and Lamin B1 were used as cytoplasmic and nuclear loading controls, respectively.

Immunofluorescences assay

Cells were seeded in 24-well plates and allowed to adhere overnight. After treatment, cells were fixed with 4% paraformaldehyde for 15 min at room temperature, permeabilized with 0.1% Triton X-100 in PBS for 10 min, and blocked with 5% bovine serum albumin (BSA) in PBS for 1 h. For F-actin staining, cells were incubated with phalloidin–Alexa Fluor 488 (1:200; Invitrogen, Carlsbad, CA, USA) for 1 h at room temperature in the dark. Nuclei were counterstained with 4' ,6-diamidino-2-phenylindole (DAPI; 1 μg/mL; Sigma-Aldrich, St. Louis, MO, USA) for 5 min [3]. For β-catenin localization, cells were incubated with primary antibody against β-catenin (1:200; Cell Signaling Technology, Danvers, MA, USA) overnight at 4 °C, followed by incubation with the appropriate fluorescent secondary antibody for 1 h at room temperature in the dark. Coverslips were mounted with antifade mounting medium (Vector Laboratories, Burlingame, CA, USA), and

images were captured using a confocal laser scanning microscope (Zeiss LSM 800, Oberkochen, Germany).

For F-actin cytoskeleton analysis, cells exhibiting prominent stress fibers or filopodia were manually counted using ImageJ. At least five random fields per sample were analyzed, and the percentage of cells with obvious stress fibers or filopodia was calculated as the number of positive cells divided by the total number of cells (DAPI-stained nuclei) × 100. For β-catenin nuclear translocation analysis, the fluorescence intensity of β-catenin in the nucleus and cytoplasm was quantified using ImageJ. The nuclear-to-cytoplasmic (N/C) ratio of β-catenin was calculated to evaluate its translocation.

IHC staining

Tissue specimens were fixed in 4% paraformaldehyde, decalcified in 10% EDTA, embedded in paraffin, and sectioned at 5-μm thickness. Tissue sections were deparaffinized, rehydrated, and subjected to antigen retrieval in citrate buffer (10 mM, pH 6.0) at 95 °C for 20 min. Endogenous peroxidase activity was quenched with 3% hydrogen peroxide, and nonspecific binding was blocked with 5% bovine serum albumin (BSA). Slides were then incubated overnight at 4 °C with primary antibodies against NTN-1, N-cadherin, Vimentin, and ZO-1. After washing, sections were incubated with HRP-conjugated secondary antibodies, developed using a DAB substrate kit, counterstained with hematoxylin, dehydrated, mounted, and visualized under a light microscope. All tissue sections were processed under identical experimental conditions and analyzed using ImageJ software. IHC staining was evaluated using a semi-quantitative H-score method. Staining intensity was classified as 0 (negative), 1 (weak), 2 (moderate), or 3 (strong). The percentage of positively stained cells at each intensity level was determined, and the H-score was calculated using the formula: H-score = $\Sigma (P_i \times I_i)$ where Pi represents the percentage of cells stained at each intensity (0–100%), and Ii represents the staining intensity (0–3) [4]. All slides were independently evaluated and the average score was used for statistical analysis.

ELISA assay

The levels of target NTN-1 proteins in cell culture supernatants or tissue lysates were quantified using commercially available ELISA kits according to the manufacturer's instructions. Briefly, samples and standards were added to 96-well plates pre-coated with specific antibodies and incubated at 37 °C for 1–2 h. After washing, biotinylated detection antibodies were added, followed by incubation with HRP-conjugated streptavidin. The reaction was developed using tetramethylbenzidine (TMB) substrate and stopped with stop solution. Absorbance was measured at 450 nm using a microplate reader. Protein concentrations were calculated based on the standard curve.

Supplementary Table S1: The primers were applied in this study

Gene	Species	Primers (5'-3')
NTN-1	Human	Forward: ACAACCCGCACAACCTGAC<br>Reverse: GGGACAGTGGAGGCGTGAC
VIM	Human	Forward: AGGCAAAGCAGGAGTCCACTGA<br>Reverse: ATCTGGCGTTCCAGGGACTCAT
CDH2	Human	Forward: CCTCCAGAGTTTACTGCCATGAC<br>Reverse: GTAGGATCTCCGCCACTGATTC
TJP1	Human	Forward: CAGCCGGTCACGATCTCCT<br>Reverse: TCCGGAGACTGCCATTGC
TNSF11A	Human	Forward: GGGTATGAGAACTTGGGATT<br>Reverse: CACTATTAATGCCACCGAC
TNF-a	Human	Forward: CCTCTCTCTAATCAGCCCTCTG<br>Reverse: GAGGACCTGGGAGTAGATGAG
IL-1β	Human	Forward: ATGATGGCTTATTACAGTGGCAA<br>Reverse: GTCGGAGATTCGTAGCTGGA
IL-6	Human	Forward: AGACAGCCACTCACCTCTTCAG<br>Reverse: TTCTGCCAGTGCCTCTTTGCTG
GAPDH	Human	Forward: ACCACAGTCCATGCCATCAC<br>Reverse: TCCACCACCCTGTTGCTGTA
NTN-1	Mouse	Forward: GAGAACGAAGACGACTCGGAG<br>Reverse: GCCGTGTTGTGCCTACAGT
VIM	Mouse	Forward: GCTGCGAGAGAAATTGCAGGA<br>Reverse: CCACTTTCCGTTCAAGGTCAAG
CDH2	Mouse	Forward: ATAGCCCGGTTTCACTTGAGA<br>Reverse: CAGGCTTTGATCCCTCTGGA
TJP1	Mouse	Forward: GCTTTAGCGAACAGAAGGAGC<br>Reverse: TTCATTTTTCCGAGACTTCACCA
TNSF11A	Mouse	Forward: CATCTTCGGCGTTTACTACAGG<br>Reverse: TCCACTTAGACTACTGCAAGCA
TNF-a	Mouse	Forward: CAAAG GGAGA GTGGT CAGGT<br>Reverse: GGCAA CAAGG TAGAG AGGC
IL-1β	Mouse	Forward: GCAACTGTTCCTGAACTCAACT<br>Reverse: ATCTTTTGGGGTCCGTCAACT
IL-6	Mouse	Forward: GATGGTCTTGGTCCTTAGCC<br>Reverse: GGGAAATCGTGGAAATGAGA
GAPDH	Mouse	Forward: TGTGTCCGTCGTGGATCTGA<br>Reverse: TTGCTGTTGAAGTCGCAGGAG

Supplementary Table S2: The concentrations of antibodies and staining dye were used in this study

Antibodies/staining dye	Source	Catalog no	Working concentrations
**β-Actin**	Santa Cruz Biotechnology, Dallas, TX, USA	SC-47778	**WB:** 1:10000
**NTN-1**	Santa Cruz Biotechnology, Dallas, TX, USA	SC-518135	**WB:** 1:1000<br>**IHC:** 1:300
**Vimentin**	Santa Cruz Biotechnology, Dallas, TX, USA	SC-6260	**WB:** 1:1000<br>**IHC:** 1:300
**N-Cadherin**	Abcam, Waltham, Massachusetts, USA	ab76057	**WB:** 1:1000<br>**IHC:** 1:300
**ZO-1**	Santa Cruz Biotechnology, Dallas, TX, USA	SC-33725	**WB:** 1:1000<br>**IHC:** 1:300
**p-GSK3β**	Santa Cruz Biotechnology, Dallas, TX, USA	SC-81496	**WB:** 1:1000
**GSK3β**	Santa Cruz Biotechnology, Dallas, TX, USA	SC-9166	**WB:** 1:1000
**p-β-Catenin**	Cell signaling	2009S	**WB:** 1:1000
**β-Catenin**	Santa Cruz Biotechnology, Dallas, TX, USA	SC-133240	**WB:** 1:1000<br>**IF:** 1:200
**Lamin B1**	Santa Cruz Biotechnology, Dallas, TX, USA	SC-56144	**WB:** 1:1000
**GAPDH**	Santa Cruz Biotechnology, Dallas, TX, USA	SC-365062	**WB:** 1:1000
**E-Cadherin**	Abcam, Waltham, Massachusetts, USA	ab40772	**WB:** 1:1000
**ERα**	Santa Cruz Biotechnology, Dallas, TX, USA	SC-8002	**WB:** 1:1000

**PR**	Santa Cruz Biotechnology, Dallas, TX, USA	SC-539	**WB:** 1:1000
**HER2**	Santa Cruz Biotechnology, Dallas, TX, USA	SC-393712	**WB:** 1:1000
**Phalloidin Alexa Fluor™ 488**	Thermofisher	A12379	**IF:** 1: 200
**Rabbit anti-Mouse IgG (H+L) Cross-Adsorbed Secondary Antibody, Alexa Fluor™ 488**	Thermofisher	A11059	**IF:** 1: 2000
**DAPI**	Sigma-Aldrich, St. Louis, MO, USA	MBD0015-1ML	**IF:** 1:3000

References

1. Law YY, Tran NB, Song CY, Wu YY, Chen HT, Fong YC, et al. Antcin K inhibits chondrosarcoma motility by reducing MMP-7 expression via downregulation of the PI3K, Akt, mTOR and NF-kappaB signaling pathway. Mol Med Rep. 2025; 32.
2. Law YY, Rengamanar H, Wu CY, Liaw CC, Ghule SS, Wu YY, et al. Ugonin P mitigates osteolytic bone metastasis by suppressing MDK via upregulating miR-223-3p expression. Int J Biol Sci. 2025; 21: 3740-54.
3. Liu CL, Ho TL, Fang SY, Guo JH, Wu CY, Fong YC, et al. Ugonin L inhibits osteoclast formation and promotes osteoclast apoptosis by inhibiting the MAPK and NF-kappaB pathways. Biomed Pharmacother. 2023; 166: 115392.
4. Jensen K, Krusenstjerna-Hafstrom R, Lohse J, Petersen KH, Derand H. A novel quantitative immunohistochemistry method for precise protein measurements directly in formalin-fixed, paraffin-embedded specimens: analytical performance measuring HER2. Mod Pathol. 2017; 30: 180-93.
